# An Autologous Macrophage‐Based Phenotypic Transformation‐Collagen Degradation System Treating Advanced Liver Fibrosis

**DOI:** 10.1002/advs.202306899

**Published:** 2023-12-08

**Authors:** Bo‐Wen Duan, Yan‐Jun Liu, Xue‐Na Li, Meng‐Meng Han, Hao‐Yuan Yu, He‐Yuan Hong, Ling‐Feng Zhang, Lei Xing, Hu‐Lin Jiang

**Affiliations:** ^1^ State Key Laboratory of Natural Medicines China Pharmaceutical University Nanjing 210009 China; ^2^ Jiangsu Key Laboratory of Druggability of Biopharmaceuticals China Pharmaceutical University Nanjing 210009 China; ^3^ Jiangsu Key Laboratory of Drug Discovery for Metabolic Diseases China Pharmaceutical University Nanjing 210009 China; ^4^ NMPA Key Laboratory for Research and Evaluation of Pharmaceutical Preparations and Excipients China Pharmaceutical University Nanjing 210009 China

**Keywords:** advanced hepatic fibrosis, hepatic stellate cells, inflammation, in situ collagen degradation, lipid nanoparticle, macrophages

## Abstract

In advanced liver fibrosis (LF), macrophages maintain the inflammatory environment in the liver and accelerate LF deterioration by secreting proinflammatory cytokines. However, there is still no effective strategy to regulate macrophages because of the difficulty and complexity of macrophage inflammatory phenotypic modulation and the insufficient therapeutic efficacy caused by the extracellular matrix (ECM) barrier. Here, AC73 and siUSP1 dual drug‐loaded lipid nanoparticle is designed to carry milk fat globule epidermal growth factor 8 (MFG‐E8) (named MUA/Y) to effectively inhibit macrophage proinflammatory signals and degrade the ECM barrier. MFG‐E8 is released in response to the high reactive oxygen species (ROS) environment in LF, transforming macrophages from a proinflammatory (M1) to an anti‐inflammatory (M2) phenotype and inducing macrophages to phagocytose collagen. Collagen ablation increases AC73 and siUSP1 accumulation in hepatic stellate cells (HSCs) and inhibits HSCs overactivation. Interestingly, complete resolution of liver inflammation, significant collagen degradation, and HSCs deactivation are observed in methionine choline deficiency (MCD) and CCl_4_ models after tail vein injection of MUA/Y. Overall, this work reveals a macrophage‐focused regulatory treatment strategy to eliminate LF progression at the source, providing a new perspective for the clinical treatment of advanced LF.

## Graphical abstract

AC73 and siUSP1 dual drug‐loaded lipid nanoparticle carrying milk fat globule epidermal growth factor 8 (MFG‐E8) (named MUA/Y) can significantly induce the macrophages phenotype transformation and effectively inhibit the source of inflammatory cytokines. Meanwhile, MUA/Y inhibits the HSCs proliferation and activation and ablates the deposited ECM to treat advanced liver fibrosis.

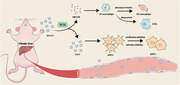

## 1 Introduction

Liver fibrosis (LF) is a self‐limiting tissue repair process caused by liver injury^[^ ^1^ ^]^ that can progress to cirrhosis and liver cancer without intervention,^[^ ^2^ ^]^ resulting in 1 million deaths worldwide each year.^[^ ^3^ ^]^ According to the lack of early‐warning symptoms, most patients are diagnosed at middle or advanced stages.^[^ ^4^ ^]^ LF is characterized by liver inflammatory infiltration,^[^ ^5^ ^]^ which is accompanied by overactivated hepatic stellate cells (HSCs)^[^ ^6^ ^]^ and extracellular matrix (ECM) deposition.^[^ ^7^ ^]^ Although many HSCs regulating strategies have been developed thus far,^[^ ^8^ ^]^ the role of inflammation in LF deterioration cannot be ignored, which leads to continuous activation of HSCs and subsequent collagen deposition,^[^ ^9^ ^]^ resulting in advanced LF that cannot be completely cured. Therefore, there is an urgent need for researchers to seek an effective method to cure advanced LF and stop the lesions from progressing in an irreversible direction.

Inflammatory infiltration is one of the characteristics of LF and occurs in almost each fibrosis pathogenesis.^[^ ^10^ ^]^ Macrophages, especially bone marrow macrophages that are recruited to the liver during inflammation, act a central role in the secretion of inflammatory cytokines.^[^ ^11^ ^]^ After being stimulated by damage‐associated molecular patterns (DAMPs) released during liver injury, macrophages are polarized into a proinflammatory (M1) phenotype and release numerous inflammatory cytokines, such as tumor necrosis factor‐α (TNF‐α), interleukin‐6 (IL‐6), and interleukin‐12 (IL‐12).^[^ ^12^ ^]^ The increased abundance of these inflammatory cytokines maintain the liver inflammatory environment and accelerate the subsequent progression of LF.^[^ ^13^ ^]^ To sever the connection between macrophages and LF deterioration, recent therapeutic strategies have achieved favorable curative effects by promoting macrophage apoptosis or depleting macrophages.^[^ ^14^ ^]^ However, as an important liver nonparenchymal cells types, macrophages are a double‐edged sword. It has been proven that macrophages can protect the liver from damage during liver failure.^[^ ^15^ ^]^ Excessive depletion of macrophages can affect liver homeostasis and cause the liver to lose its resistance to acute injury. Therefore, inducing macrophage phenotypic transformation to reduce inflammatory cytokine production is a more effective and mild strategy. It has been proven that an increase in anti‐inflammatory (M2) macrophages can inhibit the production of inflammatory cytokines and reduce tissue damage.^[^ ^16^ ^]^ However, the barrier formed by excessive ECM deposition in advanced LF prevents deep drug penetration^[^ ^17^ ^]^ and causes insufficient phenotypic transformation.^[^ ^18^ ^]^ Meanwhile, excessive ECM deposition and ECM cross‐linking prevents drug delivery to HSCs and leads to the resistance of ECM degradation, which reduces the therapeutic effect of advanced LF treatment.^[^ ^19^ ^]^ In our previous studies, various strategies have been developed to degrade collagen, such as the delivery of collagenase^[^ ^20^ ^]^ or RNA.^[^ ^21^ ^]^ However, compared with the delivery of exogenous anti‐collagen substances, it is more attractive to use autologous cells with strong phagocytic abilities to act as scavengers due to the unparalleled stability and safety of endogenous substances.^[^ ^22^ ^]^ Thus, macrophages, which are enriched in pathological environments and have considerable phagocytic capacity,^[^ ^23^ ^]^ can be trained to remove the ECM barrier, which can effectively increase drug accumulation in activated HSCs.

**Scheme 1:**
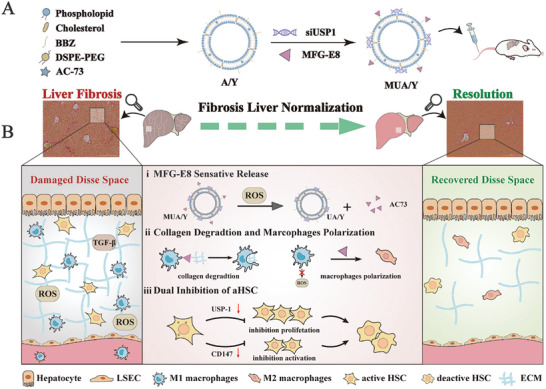
Schematic illustration of the MUA/Y lipid nanoparticles. A)The formation of A/Y and MUA/Y lipid nanoparticles. B) Under high ROS conditions in vivo, i) MFG‐E8 was released ii) and subsequently induced macrophage phenotype transformation and phagocytosis of collagen, iii) then AC73 and siUSP‐1 inhibited HSCs activation and proliferation, thereby exerting an anti‐LF effect.

Herein, we proposed a macrophage‐focused regulatory treatment strategy for remodeling the liver inflammatory environment to effectively treat advanced LF. Specifically, we constructed an AC73 and siUSP1 dual drug‐loaded lipid nanoparticles and adsorbed milk fat globule epidermal growth factor 8 (MFG‐E8) protein on the surface to form MUA/Y NPs (**Scheme** **1**). In the fibrotic region, MFG‐E8 was released in response to high reactive oxygen species (ROS) concentrations, inducing M1 macrophages to differentiate into the M2 phenotype, thereby blocking inflammation generation and inhibiting the stimulation of HSCs. Moreover, MFG‐E8 induced macrophages to phagocytose collagen via integrins, increasing drug accumulation in HSCs. AC73 is a cluster of differentiation‐147 (CD147)‐specific inhibitor,^[^ ^24^ ^]^ that can act on activated HSCs that highly express CD147 to reduce their activation; siUSP1 can inhibit the Chemokine (C‐X‐C motif) Ligand 1 Protein (CXCL1) pathway^[^ ^25^ ^]^ and further reduce HSCs proliferation. MUA/Y exerted efficient therapeutic effects on both methionine choline deficiency (MCD) and CCl_4_ models, and the mouse liver was restored to a normal status after tail vein injection of the treatment. This strategy resolved liver inflammation and ECM homeostasis simultaneously by regulating macrophages, while reshaping the HSCs phenotype and ultimately reversing fibrosis, providing a reliable approach for the treatment of advanced LF.

## 2 Results and Discussion

### 2.1 MiMFG‐E8 Drives Macrophage Phenotypic Switching

**Figure 1:**
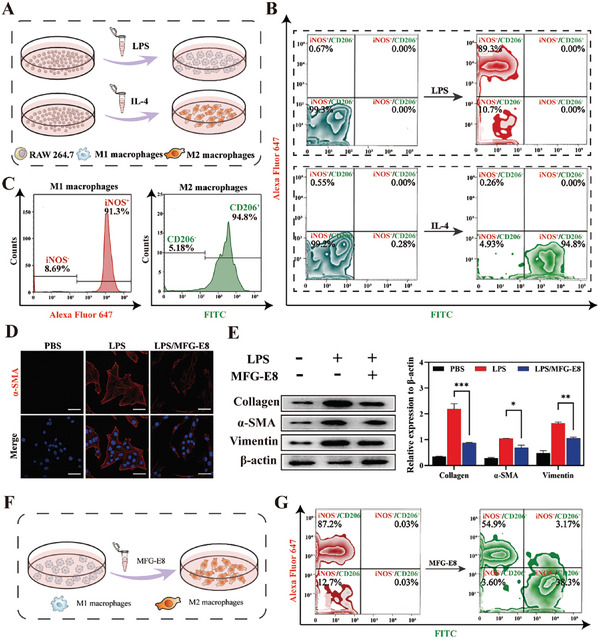
Macrophages influence the progression of LF. A) The process of M1 and M2 macrophages induced by LPS and IL‐4. B‐C) Detection of iNOS and CD206 expression in M1 and M2 macrophages by flow cytometry. D) CLSM images of immunofluorescence staining of α‐SMA of HSC after activated by M1 macrophage supernatant. Scale bar = 20 µm. E) Western blot analysis of α‐SMA in HSCs induced by M1 macrophages supernatant. The expression of collagen I, α‐SMA, and vimentin were normalized to the expression of β‐actin (*n* = 3). F) The process of M1 macrophage phenotype reversion by MFG‐E8. G) Detection of iNOS and CD206 expression by flow cytometry after incubation with MFG‐E8. Data represented mean ± SD, *p < 0.05, ***p < 0.001.

During the progression of LF, proinflammatory macrophages, which are differentiated from bone marrow‐derived monocytes, migrate to the liver and dominate the liver macrophage population.^[^ ^8c^ ^]^ However, with LF regression, these proinflammatory macrophages (M1) gradually transform into an anti‐inflammatory (M2) phenotype, which reduces inflammation. We found that the proportion of M1 macrophages was consistently the highest in the advanced fibrotic liver induced by MCD diets (Figure S1, Supporting Information), which may be due to long‐term external stimuli hindering spontaneous liver macrophage phenotypic transformation. Therefore, lipopolysaccharides (LPS) and RAW264.7 cells were cocultured to obtain M1 macrophages in vitro to simulate the association of M1 macrophages and LF progression in vivo (**Figure** **1**A). Similarly, M2 macrophages were induced by interleukin‐4 (IL‐4). Flow cytometric analysis and laser confocal microscope (CLSM) images of induced nitrogen monoxide synthase (iNOS) and cluster of differentiation‐206 (CD206), which are the biomarkers on the macrophage membrane showed that M1 and M2 macrophages were successfully induced (Figure 1B; Figure S2A, Supporting Information). The statistical results were shown in Figure 1C.

During the progression of LF, M1 macrophages are closely related to the fate of HSCs. There is an evidence that inflammatory cytokines secreted by M1 macrophages can directly stimulate HSCs activation and collagen deposition. Notably, we found that even M1 macrophage supernatant could significantly activate the HSCs (Figure S2B, Supporting Information), which was accompanied by the expression of α‐smooth muscle actin (α‐SMA) and collagen. However, the supernatant obtained from macrophages incubated with MFG‐E8 showed significantly inhibition of HSCs activation (Figure 1D), which was characterized by decreased expression of collagen and vimentin (Figure 1E), suggesting that MFG‐E8 could effectively reduce macrophage stimulation of HSCs and reduce HSCs overactivation. This may be associated with MFG‐E8 decreased the expression of the inflammatory cytokine via inhibited the p‐STAT3 pathway.^[^ ^26^ ^]^ To determine whether HSCs deactivation by MFG‐E8 as associated with a decrease in inflammatory cytokine secretion. RAW264.7 cells primed with LPS were treated with MFG‐E8 (Figure 1F). Flow cytometric analysis of iNOS and CD206 expression showed that the proportion of M1 macrophages decreased from 87.2% to 54.9% and M2 macrophages increased to 38.3% after MFG‐E8 administration (Figure 1G; Figure S2C, Supporting Information). These results suggest that MFG‐E8 drives the macrophages' phenotypic transformation.

To investigate whether MFG‐E8 could induce phenotypic transformation in macrophages in the fibrotic liver (Figure S2D, Supporting Information). We obtained primary macrophages from the livers of MCD diet fed mice at 5 w and 9 w. After being cultured with MFG‐E8, primary macrophages underwent transformation, similar to RAW264.7 cells induced in vitro. However, only 26.5% of macrophages in early LF (5 w feeding) and 16.1% of macrophages in advanced LF (9 w feeding) were effectively transformed (Figure S2E, Supporting Information). The statistical results are shown in Figure S2F (Supporting Information). This may be related to the difficulty of phenotypic transformation caused by an abundance of M1 macrophages in advanced LF. Therefore, we urgently need to seek an efficient MFG‐E8 conversion strategy to solve the fundamental problem of LF deterioration caused by inflammatory stimulation.

### 2.2 Key Signals in HSC Proliferation and Activation

**Figure 2:**
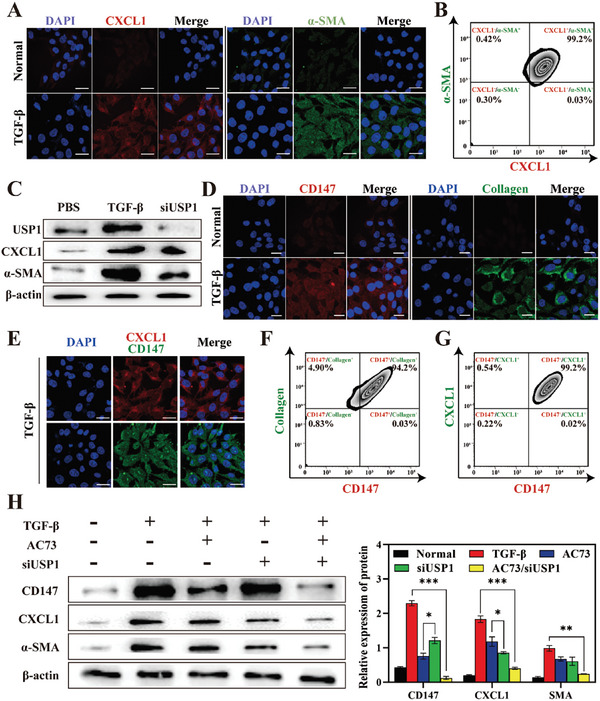
Expression of CXCL1 and CD147 in activated HSCs. A) CLSM images of CXCL1 (red) and α‐SMA (green) immunostaining in activated and quiet HSCs. Scale bars = 10 µm. B) Detection of CXCL1 and SMA expression in activated HSCs by flow cytometry. C) Western blot analysis of USP1, CXCL1, and α‐SMA in HSCs after undergoing different treatments. (*n* = 3, mean ± SD). D) CLSM images of CD147 (red) and collagen I (green) immunostaining in activated and quiet HSCs. Scale bars = 10 µm. E) CLSM images of CXCL1 (red) and CD147 (green) immunostaining in HSCs after being stimulated by TGF‐β. Scale bars = 10 µm. F,G) Detection of CXCL1/collagen I and CD147/CXCL1 co‐expression in activated HSCs by flow cytometry. H) Western blot analysis of CD147, CXCL1, and α‐SMA in HSCs after undergoing different treatments. The expression of CD147, CXCL1, and α‐SMA were normalized to the expression of β‐actin. (*n* = 3). Data represented mean ± SD, *p < 0.05, **p < 0.01, ***p < 0.001.

An increase in inflammatory cytokine secretion maintains the liver inflammatory microenvironment, and cytokine overexpression stimulates HSCs differentiation into fibroblasts, which have proliferative capacity and produce collagen, which promotes the deterioration of LF. Ubiquitin‐specific protease 1 (USP1) is a member of the deubiquitinating enzyme family, is highly expressed during HSC activation, and upregulates CXCL1 expression via de‐ubiquitination (**Figure** **2**A). Similarly, an increased proportion of α‐SMA/CXCL1 double‐positive cell populations was observed by flow cytometry (Figure 2B) while normal HSCs were α‐SMA/CXCL1 double‐negative cell populations (Figure S3A, Supporting Information). CXCL1 is widely recognized as a signal that stimulates proliferation and migration in HSCs.^[^ ^27^ ^]^ Small interfering RNA (siRNA) was used to specifically knock out USP1 to reduce CXCL1 expression and inhibit HSC proliferation. Surprisingly, we found that the expression of USP1 was significantly inhibited at the protein level, while the inhibitory effect on CXCL1 and α‐SMA was not significant (Figure 2C). The statistical results are shown in Figure S3B (Supporting Information), semiquantitative analysis showed that siUSP1 downregulated CXCL1 expression ≈35% and α‐SMA expression by 27%. These results indicated that CXCL1 expression was compensatively increased after USP1 inhibition. CD147 is a dimerized transmembrane glycoprotein and studies have shown that the positive rate of CD147 in the liver in patients with cirrhosis is 95.2%.^[^ ^28^ ^]^ During HSC activation, transforming growth factor‐β (TGF‐β) induces CD147 overexpression on the HSCs membrane, and CD147 can further increase TGF‐β expression via the transcription factor Sp1 to form a positive feedback, further aggravating LF. AC73, a specific small molecule inhibitor of CD147, can effectively reduce the expression of CD147 in HSCs, thereby reducing TGF‐β production to halt HSC activation. Fortunately, we found that AC73 reduced CXCL1 expression (Figure S3C, Supporting Information), indicating that the USP1‐CXCL1 axis intersects with the CD147‐TGF axis. CD147 may be responsible for the compensatory increase in CXCL1. Immunofluorescence staining showed high expression of CD147 and CXCL1 in activated HSCs (Figure 2E). Similarly, a CD147/CXCL1 double‐positive cell population was observed by the flow cytometry (Figure 2F), which coincided with collagen‐positive cells (Figure 2G), indicating that CD147 and CXCL1 were closely related to HSC activation and proliferation. Therefore, the combination of siUSP1 and AC73 could simultaneously influence HSCs fate, to effectively curb the deterioration of fibrosis. As shown in Figure 2H, the AC73/siUSP1 group exhibited the lowest α‐SMA expression, indicating the considerable therapeutic effect of the codelivery strategy. Semiquantitative analysis results were shown in Figure 2H. However, the electronegativity of siUSP1 and the strong hydrophobicity of AC73 limit their application in vivo, and so the development of an effective codelivery platform is a key issue in the next part of this study.

### 2.3 Synthesis and Characterization of Liposomes

**Figure 3:**
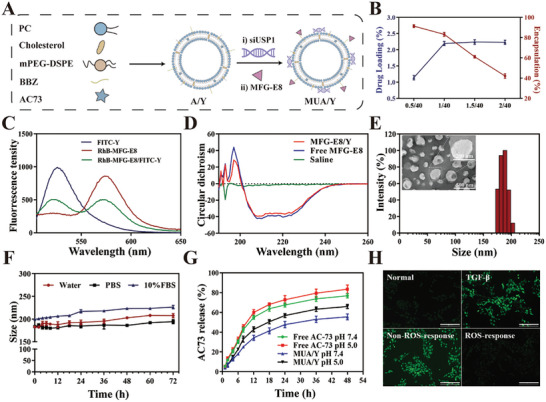
Characterization of MUA/Y lipid nanoparticles. A) The illustration on the formation of A/Y and MUA/Y. B) The DLE% and EE% of AC73 in MUA/Y lipid nanoparticles at different drug/phospholipid ratios (*n* = 3). C) The fluorescent spectrum of FITC excited RhB treated with RhB‐MFG‐E8@FITC/Y lipid nanoparticles. D) The structural changes of MFG‐E8 were verified by circular dichroism (CD). E) Size distribution and TEM result of MUA/Y. TEM Scale bar = 500 nm. F) In vitro colloidal stability of MUA/Y in water, PBS, and FBS for 72 h (*n* = 3). G) Time‐dependent AC73 release of free AC73 and MUA/Y in PBS at pH 5.0, and 7.4 (*n* = 3). H) ROS response ability of MUA/Y lipid nanoparticles. Scale bars = 100 µm. Data were represented as mean ± SD.

Lipid nanoparticles have been widely used to treat LF due to their excellent hydrophobic drug delivery capacity. MUA/Y was prepared by the thin film dispersion‐adsorption method based on our previous study.^[^ ^23^ ^]^ AC73 was encapsulated in the lipid bilayer due to its hydrophobicity. In addition, inspired by phospholipids, we added an additional carbon long‐chain compound with electropositivity and ROS responsiveness, named BBZ and the synthetic process was shown in Figure S4 (Supporting Information), non‐ROS‐responsiveness long carbon chain named BBZN and the synthetic process was shown in Figure S10 (Supporting Information). ^1^H‐NMR analysis of BBZ1‐BBZ5, and BBZN1‐BBZN3 were shown in Figures S5–S9 and Figures S11–S13 (Supporting Information), respectively. The positive charge and thioketin units on BBZ can mediate MUA/Y gene‐carrying and ROS response capabilities (**Figure** **3**A). Different ratios of AC73 to phospholipids (w/w) were used to optimize the drug loading efficiency (DLE%) and encapsulation efficiency (EE%) of AC73. First, we plotted the standard curve of AC73 (Figure S14A, Supporting Information) at 365 nm by ultraviolet spectrophotometry (UV). Subsequently, A/Y was prepared according to different prescriptions. As shown in Figure 3B, the DLE% of AC73 gradually increased with the encapsulation of AC73 and peaked when the feed ratio was 1.5:40. However, the EE% showed a continuous decreasing trend, indicating that it had reached the extreme value of liposome capacity. Similarly, the fluctuation in particle size demonstrated the instability of the nanoparticles with a feed ratio of 2:40 (Figure S14B, Supporting Information). As a result, a feed ratio of 1.5:40 (AC73: phospholipid, w/w) was chosen to prepare A/Y, of which the DLE% and EE% of AC73 were 2.21% and 60.8%, respectively (Figure 3B). The particle size of A/Y was ≈110 nm with a narrow distribution (Figure S14C, Supporting Information) and remained stable for 24 h (Figure S14D, Supporting Information). Then we investigated the effect of BBZ doping on gene loading capacity. With an increase in BBZ doping, the lipid nanoparticles showed strong electropositivity and the zeta potential of different BBZ/phospholipid ratios is shown in Figure S14E (Supporting Information). We found that a 2:1 feed ratio of BBZ to phospholipid could meet the gene delivery requirements (Figure S14F, Supporting Information). However, the main challenge of cationic lipid nanoparticles is their instability under physiological conditions. We further evaluated the stability of siNC/Y lipid nanoparticles against RNase and SDS. As shown in Figure S14G (Supporting Information), cationic lipid nanoparticles had excellent protective effects on siNC. These results indicate that cationic lipid nanoparticles (Y) have excellent gene delivery abilities. The zeta potential of different formulations at a BBZ/phospholipid ratio of 1:2 is shown in Figure S14H (Supporting Information).

We then verified whether the protein was successfully adsorbed by fluorescence resonance energy transfer (FRET). Figure 3C shows that after the excitation light of fluoresceine isothiocyanate (FITC) was given, the emission peak of rhodamine B (Rh B) at 580 nm significantly increased, while that of FITC at 520 nm decreased, revealing the energy transfer from FITC to Rh B, which proved that MFG‐E8 was adsorbed on the surface of the lipid nanoparticles. Moreover, circular dichroic chromatography (CD) showed that the structure of MFG‐E8 was not damaged after adsorption (Figure 3D). The particle size of the final preparation MUA/Y, which was formed after the adsorption of siUSP1 and MFG‐E8 was ≈175 nm, had a narrow distribution and exhibited the classical opalescence of nano‐formulations. A clear white spherical structure was shown in the teansmission electron microscope (TEM) analysis of MUA/Y nanoparticles (Figure 3E), which also proved the successful adsorption of MFG‐E8. MUA/Y had an extremely high particle size stability in PBS and 10% FBS, which mimics body circulation (Figure 3F). AC73 release behavior was then further measured in PBS at pH 7.4 and 5.0, which represent physiological conditions and lysosomal chambers, respectively. The results in Figure 3G show that ≈46% of AC73 was released within 72 h at pH 7.4. Under acidic conditions, the release rate of AC73 was significantly accelerated, and at pH 5.0, the release rate is ≈65% after 72 h. This pH‐sensitive release property may be due to the protonation of the amino groups in BBZ in an acidic environment, which improves the water solubility of cationic liposomes and promotes AC73 release. Finally, the responsive cleavage of MFG‐E8 is a prerequisite for the phenotypic reversal of macrophages, and we investigated the ROS responsiveness of the lipid nanoparticles. We used DCFH‐DA as a ROS probe. As shown in Figure 3H, similar to the untreated group, cells treated with lipid nanoparticles produced no visible ROS. In contrast, cells treated with non‐ROS‐responsive polymers showed obvious green fluorescence, indicating that the thioketal units in lipid nanoparticles could consume ROS and prevent DCF from being generated through oxidation, demonstrating the excellent ROS consumption by the thioketal units in MUA/Y and the potential of inhibiting the HSCs overactivation caused by ROS.

### 2.4 MFG‐E8 Mediated Macrophages Phagocytosis of Collagen

**Figure 4:**
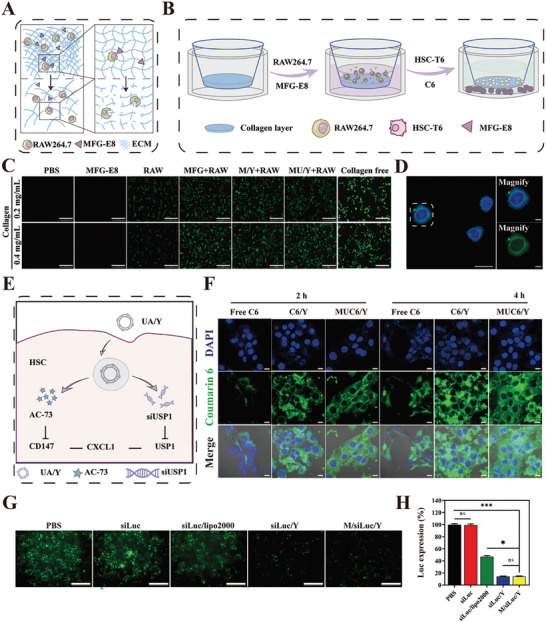
Characterization of collagen phagocytosis, cellular uptake, and endosome escape of MUA/Y lipid nanoparticles. A) The process of collagen phagocytosis mediates by MFG‐E8. B) The process of MFG‐E8 mediated macrophages ablate collagen. C) Fluorescence intensity in HSCs detected by fluorescence microscopy. Scale bars = 100 µm. D) FITC‐modified collagen was endocytosed by RAW264.7. The image was captured by CLSM. Scale bars = 10 µm. E) The process of MUA/Y endocytosed escape in HSCs. F) CLSM images of cellular uptake of free C6, C6/Y, and MUC6/Y under 2 and 4 h. Scale bars = 20 µm. G) Transfection efficiency of siLuc loaded lipid nanoparticles detected by fluorescence microscopy. Scale bars = 100 µm. H) Semiquantitative analyze of the Luc positive area of fluorescent cells. (*n* = 3). Data represented mean ± SD. ns: no significant difference, *p < 0.05, ***p < 0.001.

LF progression is typically accompanied by a large amount of collagen deposition, resulting in poor drug accumulation in the target cells and, reducing the therapeutic effect on advanced LF. The inherently powerful phagocytic ability of macrophages makes them an optimal choice for use as collagen ablators. Specifically, macrophages bind to collagen under the mediation of MFG‐E8, which promotes macrophage phagocytosis of collagen (**Figure** **4**A). To visualize the degradation of collagen by macrophages in the presence of MFG‐E8, we designed a novel experiment (Figure 4B). In short, the collagen layer and HSCs were seeded in the upper chamber and corresponding the invasion chamber. Then macrophages and MFG‐E8 were coadded to the upper compartment and removed after 4 h, and MUC6/Y (loading Coumarin 6 (C6)) was added immediately. Compared with those in the control group, HSCs showed marked fluorescence signals after RAW and MFG‐E8 were cocultured, and there was no significant difference in the fluorescence intensity of the MFG‐E8 preparation (M/Y and MU/Y). We also investigated the effect of different collagen concentrations on degradation efficiency and found that MFG‐E8 could guide macrophages to ablate different concentrations of collagen (Figure 4C) and the statistics analyze result was shown in Figure S15A (Supporting Information). To observe macrophage collagen phagocytosis, macrophages were cultured in a dish with FITC‐labeled collagen at the bottom and then observed under CLSM. In response to MFG‐E8 stimulation, macrophage cytoplasm showed an obvious green fluorescence signal (Figure 4D), indicating the efficiency of collagen endocytosis by macrophages. In addition, we found that MUA/Y did not cause changes in the phenotypic transformation efficiency of MFG‐E8 on macrophages. Flow cytometric analysis of iNOS and CD206 expression showed that the proportion of M1 and M2 macrophages were 60.9% and 34.4%, respectively, after MUA/Y administration. The reversion efficiency of MUA/Y shows no difference with free MFG‐E8 group (Figure S15B, Supporting Information). Meanwhile, the increase of M2 ratio proved that macrophages transformed to anti‐inflammatory phenotype, which led to the inhibition of macrophage supernatant on HSCs activation. (Figure S15C, Supporting Information)

### 2.5 Cellular Uptake, Endosomal Escape, and In Vivo siRNA Transfection

Effective gene delivery includes cellular uptake, endosomal escape, and cytoplasmic transport of genes (Figure 4E). C6 was used as a model drug to replace AC73, and the results showed that the uptake of lipid nanoparticles by HSCs was time dependent (Figure 4F), and the statistics analyze of C6 positive area was shown in Figure S15D (Supporting Information). There was no significant differences in the fluorescence intensity observed between C6/Y and MUC6/Y, indicating that gene and protein adsorption did not affect lipid nanoparticle internalization by cells. Flow cytometry showed no significant differences between these two groups (Figure S15E, Supporting Information). Moreover, the uptake mechanism was evaluated and showed that the lipid nanoparticles entered the cells mainly through caveolae‐mediated endocytosis (amiloride was used as an inhibitor of macropinocytosis; methyl‐β‐cyclodextrin (MβCD) was used as an inhibitor of caveolae‐mediated endocytosis; chlorpromazine was used as an inhibitor of clathrin‐based endocytosis) (Figure S15F, Supporting Information). After the liposome is endocytosed by the cell, the success of endo/lysosomal escape of the gene is the critical step of gene transfection. BBZ contains a proton response unit that can assist in endo/lysosomal escape. Lysosome and siRNA were separately labeled with red (LysoTracker) and green (FAM) fluorescence. The colocalization or separation of the endo/lysosome and siRNA was used to determine endo/lysosomal entrapment or escape. The superimposed fluorescence signal was captured in the siFAM/Y and M/siFAM/Y groups at 3 h, and the results showed that most of the lipid nanoparticles were trapped in the endo/lysosome (Figure S15G, Supporting Information). However, after another 3 h of incubation, green fluorescence was retested in each group, and the yellow signals had disappeared. At this time point, the preparation was separated from the endosomes, and the siRNA had completed its transport into the cytoplasm.

Electronegative substances such as serum can nonspecifically bind to cationic lipid nanoparticles during their circulation in vivo,^[^ ^21b^ ^]^ thus affecting gene transfection. Luciferase reporter genes were used as models to evaluate gene transfection. Compared with siLuc/Lipo2000, siLuc/Y and M/siLuc/Y showed stronger gene silencing ability in the absence of FBS, which may be caused by the stronger positivity of cationic lipid nanoparticles (Figure 4G). Statistical results of fluorescence intensity are shown in Figure 4H. There was no significant difference in fluorescence intensity between siLuc/Y and M/siLuc/Y, indicating that the additional adsorption of MFG‐E8 did not affect gene silencing by the siRNA. In the presence of FBS, the nonspecific binding between serum proteins and cationic liposomes resulted in a significant decrease in gene transfection efficiency. We compared luciferase silencing efficiency in the presence of different FBS concentrations. The fluorescence intensity increased when a higher concentration of FBS was added, indicating that excessive serum proteins could weaken the knockout efficiency of genes. However, the silencing efficiency of cationic liposomes was still considerable in normal systemic circulation (Figure S15H, Supporting Information) and the statistics analysis result was shown in Figure S15I (Supporting Information).

### 2.6 Cell Apoptosis

The toxicity of cationic liposomes is a significant hurdle associated with in vivo administration. We examined the cytotoxicity of MUA/Y to major cells in the liver (L02, RAW264.7, and HSC‐T6). As shown in Figure S16A, (Supporting Information), the viability of HSCs was negatively correlated with AC73 concentration and the A/Y group showed the lowest cell viability, which might be due to an increase in internalization by cells caused by the high electropositivity. However, the free AC73 and MUA/Y groups exhibited moderate HSC toxicity at equal AC73 concentrations, which may be due to the high hydrophobicity leading to a decrease in cellular uptake, and the charge neutralization caused by the adsorption of the negative component, which reduced toxicity. Therefore, we selected a concentration of AC73 of 2 µg mL^−1^ for follow‐up experiments, and the survival rate of HSCs in each group was higher than 75%. Similarly, the same outcome was observed in hepatocytes (L02) and macrophages (RAW 264.7), and fortunately, these cells showed a higher safety profile in the presence of MUA/Y (Figure S16B,C, Supporting Information), avoiding the damage of MUA/Y to other liver cells and causes liver dysfunction. Subsequently, we demonstrated that the MFG‐E8 protein was not toxic to liver cells (Figure S16D, Supporting Information). Furthermore, an apoptosis assay was performed to investigate each formulation's toxicity. There was no obvious cytotoxicity of MUA/Y in macrophages or HSCs. The apoptosis rate induced by MUA/Y was negligible, indicating its excellent biosafety (Figure S17, Supporting Information).

### 2.7 MUA/Y Lipid Nanoparticles Inhibited the Activation of HSC

**Figure 5:**
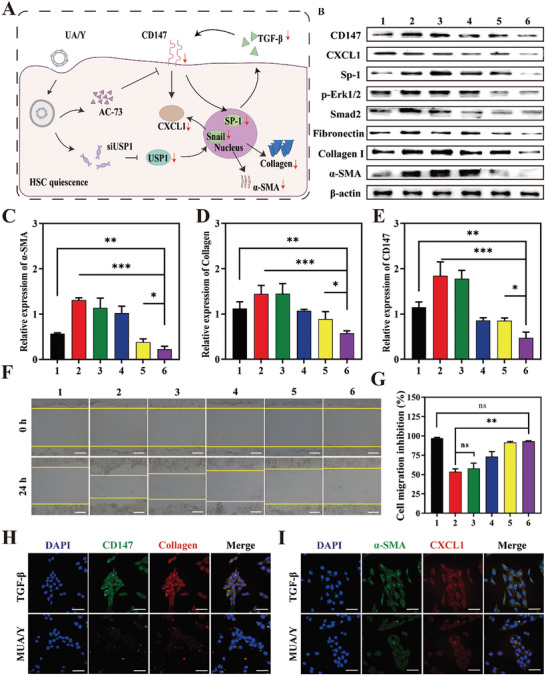
Mechanisms of MUA/Y lipid nanoparticles in HSCs. A) Mechanisms of HSCs activation and recovery after treatment. B) The expression of CXCL1, CD147, Sp‐1, Erk1/2, Smad2, fibronectin, collagen I, and α‐SMA in HSCs undergoing different formulations. 1 means PBS group, 2 means TGF group, 3 means free AC73 group, 4 means siUSP1/Y, 5 means AC/Y, 6 means UA/Y. C–E) Densitometric quantification of western blotting results in each group. The expression of α‐SMA, collagen I and CD147 were normalized to β‐actin. (*n* = 3). F) Images for cell scratch assay of HSCs at 0 and 24 h (*n* = 3). Scale bars = 100 µm. G) Semi‐quantified of the scratch area of each group, the scratch area in each groups were normalized to PBS group (*n* = 3). H,I) CLSM images of CD147 (green)/CoI (red) and SMA (green)/CXCL1 (red) immunostaining in HSCs after treatment (*n* = 3). Scale bars = 100 µm. Data were represented as mean ± SD, ns: no significant difference, *p < 0.05, **p < 0.01, ***p < 0.001.

AC73 and siUSP1 were released after MUA/Y was internalized by HSCs. At this time, AC73 specifically triggered the inhibition of CD147 dimerization which was overexpressed on the surface of HSCs, further disrupting the SP1‐mediated CD147‐SP1‐TGF cycle and reducing the expression of CXCL1. Moreover, the Snail‐CXCL1 pathway was inhibited by siUSP1, which reduced CXCL1 expression (**Figure** **5**A). We evaluated the potential synergistic effect of AC73 and siUSP1 in this context by using TGF‐β to stimulate quiescent HSCs and detected the expression of fibrosis‐associated proteins. The combination of AC73 and siUSP1 in MUA/Y showed the best antifibrotic effect, and the expression levels of fibrotic factors such as α‐SMA and collagen were decreased (Figure 5B; Figure S18, Supporting Information). In addition, the decreased expression of CD147, USP1, and CXCL1 indicated the intracellular efficacy of the two drugs. In addition to promoting HSCs proliferation, CXCL1 has been shown to be associated with HSCs migration and survival.^[^ ^29^ ^]^ Therefore, we evaluated the effect of MUA/Y on HSC migration and contraction. Cell scratches were photographed at 0 and 24 h (Figure 5F) and wound healing rates were calculated. Untreated HSCs showed healing after 24 h of culture. However, the scratch area of TGF treated cells decreased significantly after 24 h, due to the proliferation and migration of HSCs stimulated by TGF‐β. However, compared with the other groups, there was no significant change in scratch area under the combined treatment of USP1 and AC73, suggesting that siUSP1 and AC73 had a certain inhibitory effect on the proliferation and migration of TGF‐β‐induced HSCs. The scratch healing rates of UA/Y and MUA/Y were not different from those in the blank control group. Overall, the scratch experiments (Figure 5G) showed that MUA/Y‐transmitted AC73 and siUSP1 significantly inhibited HSC activation and proliferation and reduced ECM secretion. These results are consistent with those of previous studies. Furthermore, to visually verify the inhibition of HSC activation and proliferation after treatment with the different formulations, fluorescence labeling was used to observe protein expression levels. Figure 5H,I shows that CD147 and SMA were labeled with green fluorescence and CXCL1 and CoIlagen with red fluorescence. After TGF stimulation, the colocalization of CD147/CoIlagen and CXCL1/SMA fluorescence demonstrated that HSCs were activated. The disappearance of yellow fluorescence after treatment demonstrated that MUA/Y could inhibit activation and proliferation, demonstrating a promising therapeutic approach.

### 2.8 Tissue Distribution and Cellular Localization

**Figure 6:**
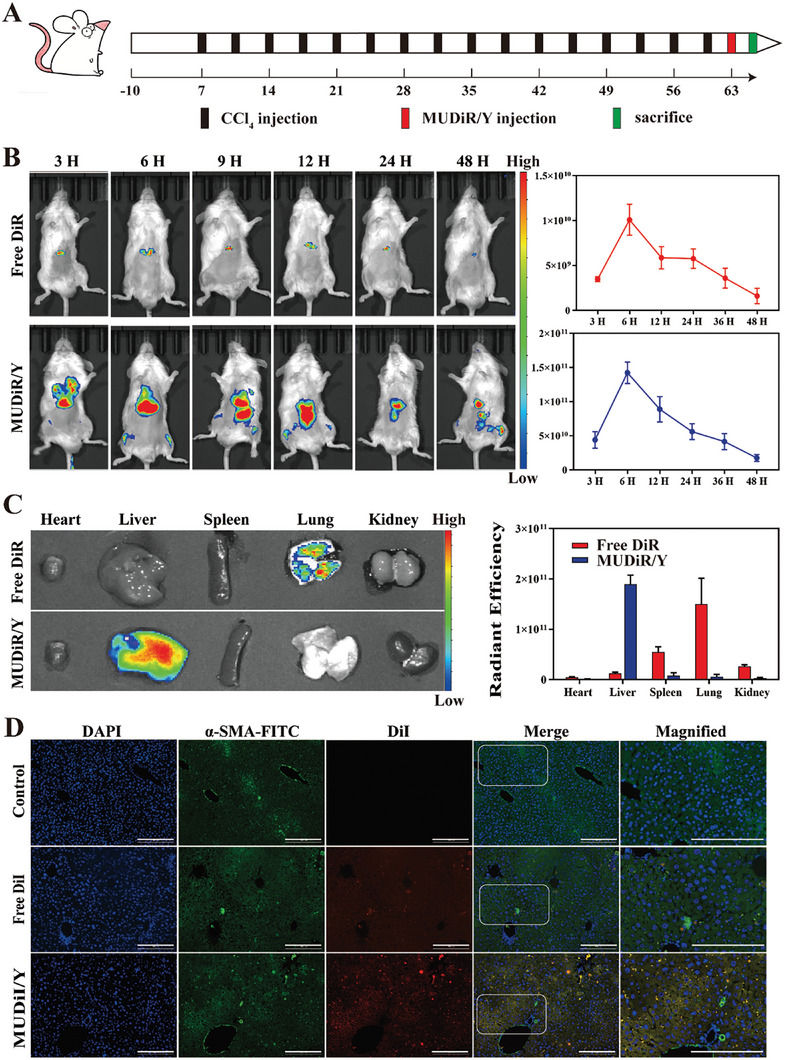
Biodistribution and Intrahepatic distribution of MUA/Y lipid nanoparticles after intravenous injection. Mice received DIR‐loaded liposomes and free DiR administration 72 h after the last CCl_4_ injection. A) Schedule of the establishment of the mice LF model. B) Images of DiR tissue distribution in fibrotic mice in different groups at the indicated time points (*n* = 3) and quantities analysis of fluorescence intensity. C) Images of DiR distribution in main organ in different groups at the indicated time points (*n* = 3) and quantities analysis of fluorescence intensity. D) Analysis of the intrahepatic distribution of the AC73 (DiI) in fibrotic liver. Scale bars = 100 µm.

Effective liver accumulation of drugs after injection is necessary for the treatment of LF. To investigate the tissue distribution of MUA/Y, free DiR or DiR‐loaded nanoparticles (MUDiR/Y) were intravenously injected into the fibrosis mice, and in vivo imaging was used to measure near‐infrared fluorescence emission for 48 consecutive hours (**Figure** **6**A). As shown in Figure 6B, the accumulation of MUDiR/Y in the liver was much higher than that of free DiR. Quantitative analysis showed that the average radiant efficiency of MUDiR/Y was 15 times higher than that of DiR (Figure 6B). At 48 h after injection into the tail vein, the fluorescence emission in the main organs was measured. In general, the amount that accumulated in the liver was much higher than that in other organs. In addition, MUDiR/Y produced a stronger signal compared free DiR, indicating that this delivery system can effectively accumulate in the liver (Figure 6C). Next, mice were injected with CCl_4_ for 9 w and then injected via the tail vein with DiI alone or loaded with lipid nanoparticles daily for 3 days. After 24 h, the liver was removed and frozen. The sections were then immunostained for α‐SMA and counterstained with DAPI. As shown in Figure 6D, the red fluorescence in mice fused with MUDiI/Y fuses with the green fluorescence of α‐SMA and further produced a yellow signal, clearly indicating that DiI was internalized by HSCs. In free DiR group and normal group, red fluorescence was much weaker, and yellow fluorescence was observed. These results clearly show that MUA/Y can efficiently accumulate in the liver and be internalized by HSCs, and the results are consistent with previous studies.

### 2.9 Antifibrotic Effects on Liver Fibrosis Induced by MCD and CCl_4_

**Figure 7:**
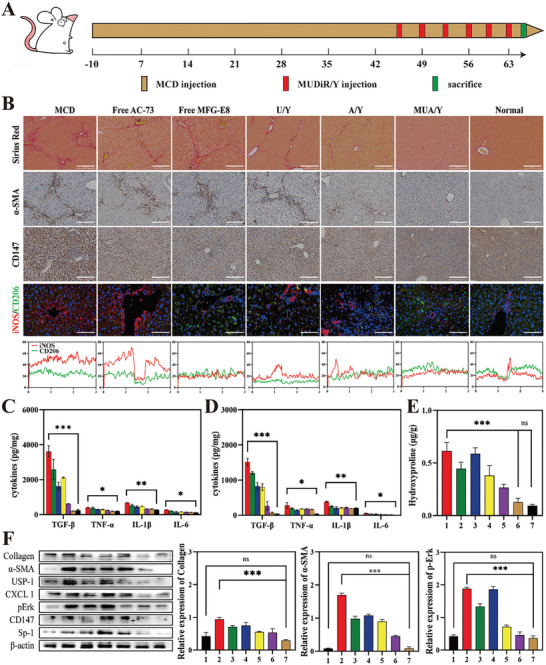
MUA/Y effectively inhibited the progression of MCD‐induced LF. A) Establishment and treatment strategy of advanced LF model. B) Representative images of Sirius red, α‐SMA, CD147 immunohistochemistry, and iNOS/CD206 immunofluorescence stained liver sections, scale bars = 100 µm. C,D) TGF‐β, IL‐1β, IL‐6, and TNF‐α levels in serum and liver tissue (*n* = 3). E) The hydroxyproline (Hyp) level in the liver of mice in each treatment group (*n* = 3). The group sequence was similar to Figure 7B. F) The expression level of Collagen I, α‐SMA, p‐Erk, CXCL1, CD147, USP1, Sp‐1 undergoing different formulation and the Semi‐quantitative analysis of Collagen I, α‐SMA, p‐Erk. 1 means normal, 2 means fibrosis group, 3 means free AC73 group, 4 means free MFG group, 5 means U/Y group, 6 means A/Y group and 7 means MUA/Y. The expression of α‐SMA, collagen I and p‐Erk were normalized to the expression of β‐actin. Data were represented as mean ± SD. *p < 0.05, **p < 0.01, ***p < 0.001, ns: no significant difference.

C57bl/6j mice were fed with a methionine choline deficiency (MCD) diet for 63 days to obtain a steatosis‐associated advanced LF model. **Figure** **7**A illustrated the treatment strategies used in the treatment of the MCD model. At 9 w, various formulations (3 mg kg^−1^ d^−1^ for AC73, 1 mg kg^−1^ d^−1^ for siUSP1, and 20 µg kg^−1^ d^−1^ for MFG‐E8) were injected intravenously. Mice's body weight was shown during the treatment period (Figure S19A, Supporting Information). The mouse body weight in the normal group increased with the extension of feeding time, while body weight in the MCD group decreased significantly. Mouse body weight in the other groups first decreased and then increased. It is possible that different treatment formulations alleviated the weight loss associated with LF. At the same time, the hepatosomatic ratio can directly reflect the condition of the liver (Figure S19B, Supporting Information). Generally, an increase in the hepatosomatic ratio indicates that the liver may have congestion or hyperplasia and hypertrophy. The increase in the hepatosomatic ratio is a classic symptom of nonalcoholic LF. We found that with the progression of treatment, the hepatosomatic ratio in the MUA/Y group gradually decreased and was not significantly different from that in the normal group. To determine the therapeutic effect of each formulation, hydroxyproline (Hyp) expression levels in hepatic tissue were detected. Hyp is a characteristic hydrolytic product of collagen, which indirectly represents collagen content in tissues. We found that the Hyp content was highest in the MCD group and decreased significantly after treatment with MUA/Y, but there was no significant difference from that in the normal group (Figure 7E). Furthermore, tissue collagen deposition was visually observed by Sirius Red staining. The results were shown in Figure 7B. Compared with that in the MCD group, the collagen content in the MUA/Y group was significantly reduced, and the liver returned to a normal state. Semiquantitative results showed the smallest collagen‐positive area in the final formulation group (MUA/Y) (Figure S19C, Supporting Information), indicating a reliable antifibrotic effect. This had a positive effect on the treatment of LF. Similarly, α‐SMA, which is an important indicator of HSC activation, can reflect the degree of fibrosis. Immunofluorescence staining showed that the α‐SMA level in the MUA/Y group was significantly decreased (Figure 7B), and semiquantitative analysis showed that it had the smallest positive area (Figure S15D, Supporting Information). Similarly, the change in CD147, which is a surface activation marker of HSCs (Figure 7B), was similar to that of α‐SMA and the semiquantitative analysis result was shown in Figure S19E (Supporting Information). To verify the effect of macrophage phenotypic transformation during treatment, we performed immunofluorescence staining to evaluate the iNOS and CD206 expression in liver sections. As shown in Figure 7B, compared with the high level of red fluorescence in the MCD group, green fluorescence increased significantly after treatment with MUA/Y, indicating the transformation of the liver from a proinflammatory state to an anti‐inflammatory state. Inflammatory cytokines, such as TGF‐β, IL‐1β, IL‐6, and TNF‐α, were detected in serum and tissues, to evaluate liver inflammatory infiltration. The results are shown in Figure 7C,D. All inflammatory cytokines in the MCD group were significantly increased and restored to normal levels after MUA/Y administration. Finally, the liver is an important organ associated with metabolism, and transaminase is an important indicator of liver function. Glutamic pyruvic transaminase (ALT) and glutamic oxaloacetic transaminase (AST) are important biomarkers that reflected the degree of fibrosis. There was no significant difference between the MUA/Y group and the PBS group, indicating that MUA/Y could effectively treat LF (Figure S19F,G, Supporting Information). Notably, there were no significant differences in the expression of creatinine (CR) and blood urea nitrogen (BUN) among the groups (Figure S20A,B, Supporting Information), which also indicated that MUA/Y had no obvious renal toxicity. Moreover, H&E‐stained sections of organs (heart, liver, spleen, lung, and kidney) in each group showed no obvious tissue necrosis after treatment (Figure S21, Supporting Information), which indicated high biocompatibility. According to the blood compatibility assays, there was no hemolysis of mice blood after treated with different concentrations of MUA/Y lipid nanoparticles (0.1–250 µg mL^−1^) (Figure S24, Supporting Information). We evaluated the therapeutic effect on protein levels. As shown in Figure 7F, collagen, and SMA were significantly decreased in the MUA/Y group, demonstrating the reversal of advanced fibrosis. The decrease in CXCL1, p‐Erk1/2, and USP1 levels indicated that the proliferation of HSCs was significantly inhibited. The semiquantitative results are shown in (Figure S19H–K, Supporting Information). The physiological markers (WBC, RBC, and Neu) in the MUA/Y lipid nanoparticle‐treated group exhibited no significant differences compared with those in the normal group (Figure S20C–I, Supporting Information). WBC levels increased significantly compared with the MCD group, indicating the relief of blood cell entrapment related to splenomegaly caused by increased portal vein pressure,^[^ ^30^ ^]^ which also demonstrated the therapeutic effect of MUA/Y. These results indicated that MUA/Y possessed good biocompatibility and might be a promising candidate for advanced LF therapy.

In addition, we also evaluated the antifibrotic effect of MUA/Y in a CCl_4_‐induced advanced LF model. Figure S22A (Supporting Information) illustrated the treatment strategy of the CCl_4_ model. Similarly, body weight, hepatosomatic ratio, and Hyp content in the CCl_4_ model all demonstrated the alleviation of advanced LF (Figure S22B–D, Supporting Information). The Sirius Red staining of collagen also indicated that collagen deposits have been effectively removed (Figure S22E, Supporting Information) and the statistics analyze of the Sirius red positive area was shown in Figure S22F (Supporting Information). Meanwhile, the serum and liver proinflammatory cytokines decreased to normal levels, indicating that MUA/Y has excellent anti‐inflammatory effects (Figure S22G,H, Supporting Information). ALT, AST, and ALP levels were no significant difference between in the MUA/Y group and the PBS group (Figure S22I–K, Supporting Information) and BUN and CR level were shown in Figure S23A,B (Supporting Information). Similar to the MCD model, there were no abnormalities in physiological indicators (RBC, WBC) detection (Figure S23C–I, Supporting Information) and H&E staining (Figure S23J, Supporting Information), indicating that MUA/Y has high biosafety. Therefore, MUA/Y has a good therapeutic effect on LF caused by a variety of pathogenesis.

## 3 Conclusion

In summary, we propose a strategy to regulate macrophages to treat advanced LF. This autologous macrophage‐based phenotypic conversion not only eliminates fibrosis caused by liver inflammation but also degrades ECM in advanced LF and effectively reduces HSC proliferation and activation, ultimately treating the symptoms and root causes of advanced LF. The results showed that 1) MUA/Y treatment could significantly induce the transformation of liver macrophages to an anti‐inflammatory phenotype and effectively inhibit the source of inflammatory cytokines. 2) MUA/Y could almost completely ablate collagen deposition in advanced LF, and there was no significant difference in collagen content compared with that in the normal group after treatment. 3) MUA/Y simultaneously inhibited the proliferation and activation of HSCs, which solved the fundamental problem in the treatment of advanced LF and had the best therapeutic effect. Our strategy involved regulating macrophages to inhibit their continuous stimulation of HSCs while effectively inhibiting HSC proliferation and activation, providing an antifibrotic treatment regimen for the pathogenesis of LF.

## Conflict of Interest

The authors declare no conflict of interest.

## Author Contributions

B.‐W.D. and Y.‐J.L. performed formulation or evolution of overarching research goals and aims. B.‐W.D. and Y.‐J.L. conducted a research and investigation process, specifically performing the experiments, or data collection. B.‐W.D., X.‐N.L., and M.‐M.H. performed preparation, creation, and presentation of the published work, specifically data presentation. B.‐W.D., H.‐Y.Y., H.‐Y.H., and L.‐F.Z. performed animals study. B.‐W.D. performed preparation and presentation of the published work, specifically writing the initial draft. H.‐L.J. and L.X. performed oversight and leadership responsibility for the research activity planning and execution.

## Supporting information

Supporting Information

## Data Availability

Research data are not shared.
