# Supplementary material for: An Autologous Macrophage‐Based Phenotypic Transformation‐Collagen Degradation System Treating Advanced Liver Fibrosis
Source: Adv Sci (Weinh). 2023 Dec 8;11(7):2306899. doi: 10.1002/advs.202306899 (PMC10870050; doi:10.1002/advs.202306899)
Supplement: Supplementary file 1 — Supporting Information [file ADVS-11-2306899-s001.pdf]

ADVANCED SCIENCE
Open Access

Supporting Information

for *Adv. Sci.*, DOI 10.1002/advs.202306899

An Autologous Macrophage-Based Phenotypic Transformation-Collagen Degradation System Treating Advanced Liver Fibrosis

*Bo-Wen Duan, Yan-Jun Liu, Xue-Na Li, Meng-Meng Han, Hao-Yuan Yu, He-Yuan Hong, Ling-Feng Zhang, Lei Xing and Hu-Lin Jiang**

Supporting Information

An Autologous Macrophage-based Phenotypic Transformation-Collagen Degradation System Treating Advanced Liver Fibrosis

Bo-Wen Duan[1], Yan-Jun Liu[1], Xue-Na Li[2], Meng-Meng Han[1], Hao-Yuan Yu[1], He-Yuan Hong[1], Ling-Feng Zhang[1], Lei Xing[1], Hu-Lin Jiang[1,2,3*]

Bo-Wen Duan, Yan-Jun Liu, Xue-Na Li, Meng-Meng Han, Hao-Yuan Yu, He-Yuan Hong, Ling-Feng Zhang, Lei Xing, Hu-Lin Jiang

State Key Laboratory of Natural Medicines, China Pharmaceutical University, 210009, China

E-mail: jianghulin3@163.com

Prof. H. L. Jiang.

1. Jiangsu Key Laboratory of Druggability of Biopharmaceuticals, China Pharmaceutical University, 210009, China
2. Jiangsu Key Laboratory of Drug Discovery for Metabolic Diseases, China Pharmaceutical University, 210009, China
3. NMPA Key Laboratory for Research and Evaluation of Pharmaceutical Preparations and Excipients, China Pharmaceutical University, 210009, China.

*Corresponding author. Email: jianghulin3@gmail.com.

Materials and Methods

Materials

Phospholipid was purchased from Avanti (Shanghai, China). Cholesterol was purchased from Sinopharm Chemical Reagent Co., Ltd (Beijing, China). DSPE-PEG_{2000} was purchased from Shanghai Yarebio Co., Ltd (Shanghai, China). AC73 was purchased from Shanghai Zixia Co., Ltd (Shanghai, China). siUSP1 and fluorescently labeled FAM-siRNA were synthesized by Genepharma Ltd (Suzhou, China). MFG-E8 was purchased from the R&D system (USA). 2',7'-Dichlorofluorescin diacetate (DCFH-DA) was purchased from Sigma-Aldrich (St. Louis, MO, USA). Dulbecco's modified eagle medium (DMEM), 3-(4, 5-dimethylthiazol-2-yl)-2, 5-diphenyltetrazolium bromide (MTT), and DAPI were purchased from KeyGEN BioTECH (Nanjing, China). Trypsin-EDTA (0.25 %) was purchased from Gibco (Burlington, Canada). DiR iodide was purchased from Fanbo Biochemical (Beijing, China). The hydroxyproline assay kit was purchased from Nanjing Jiancheng Bioengineering Institute (Nanjing, China). TGF-β was purchased from PeproTech Inc (New Jersey, USA). Anti-fibronectin, anti-vimentin, anti-CD147, anti-CXCL1 and anti-ERK1/2 antibodies were purchased from Abcam (Cambridge, UK). Anti-collagen I and anti-α-SMA antibodies were purchased from Proteintech (Wuhan, China). FITC-anti mouse CD206 and Alexa Flour 647-anti mouse iNOS were purchased from BioLegend. The rest were commercially qualified reagents.

Methods

Synthesis and characterization of BBZ-1

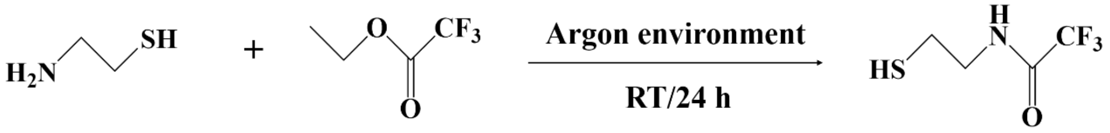

2-aminoethane-1-thiol hydrochloride (1 g, 8.85 mmol), ethyl trifluoroacetate (TFAE) (3.77 g, 26.55 mmol), and triethylamine (TEA) (2.69 g, 26.55 mmol) were added into tetrahydrofuran (30 mL). Then the mixture was added to a 100 mL round-bottom flask

and kept stirring at room temperature under an argon environment for 24 h (TLC, hexane: ethyl acetate = 3:1, v/v). The product was purified by column chromatography (hexane: ethyl acetate =6:1, v/v). The white crystal was obtained with a yield of 90%. ^{1}H-NMR (300 MHz, $CDCl_3$), δ(ppm): 3.62 (m, 2H, $NHCH_2$), 2.87-2.90 (m, 2H, SCH_2).

Synthesis and characterization of BBZ-2

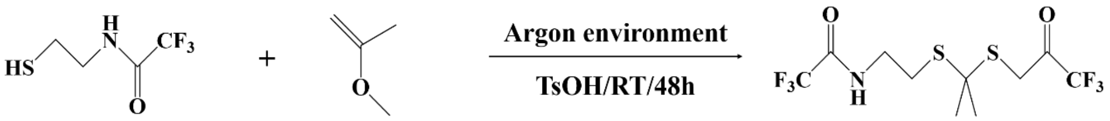

BBZ-1 (1.25 g, 7.2 mmol), 2-methoxypropylene (0.25 g, 3.5 mmol), p-Toluenesulfonic acid (TsOH) (0.125 g, 0.72 mmol) and sieve sorbent (5 g) were added into tetrahydrofuran (30 mL). Then the mixture was stirred in a 100 mL round-bottom flask under an argon environment at room temperature for 48 h (TLC, hexane: ethyl acetate = 3:1, v/v). Column chromatography purified the product (hexane: ethyl acetate =15:1, v/v). The white crystal was obtained with a yield of 87%. ^{1}H-NMR (300 MHz, $CDCl_3$), δ(ppm): 6.87 (s, 2H, 2NH), 3.62-3.64 (m, 4H, $NHCH_2$), 2.87-2.90 (m, 4H, SCH_2), 1.67 (s, 6H, $2CH_3$).

Synthesis and characterization of BBZ-3

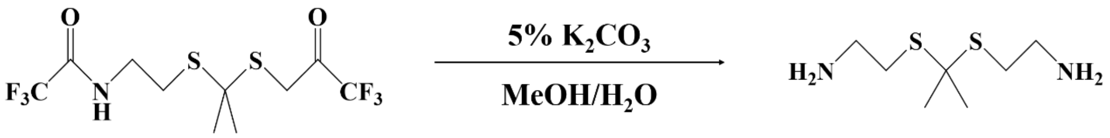

BBZ-2 (1.1 g, 2.95 mmol) was dissolved in a 50 mL round-bottom flask and then sodium hydroxide (6 mol L^{-1}, 30 mL) was dripped slowly under the stirring condition at room temperature overnight (TLC, hexane: ethyl acetate = 3:1, v/v). The product was extracted by ethyl acetate (20 mL) three times and then washed twice with saturated sodium chloride (20 mL). The organic phase was dried overnight with anhydrous sodium sulfate. A yellow oil was obtained with a yield of 85% and the product was without any other purification. ^{1}H-NMR (300 MHz, $CDCl_3$), δ(ppm): 3.62-3.64 (m, 4H, $NHCH_2$), 2.87-2.90 (m, 4H, SCH_2).

Synthesis and characterization of BBZ-4

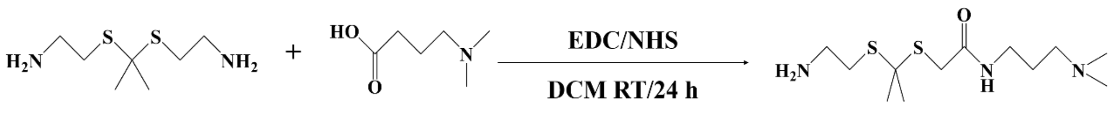

BBZ-3 (0.4 g, 2 mmol), *N*-3-dimethylpropane-1,3-diamine (EDC) (0.459 g, 2.4 mmol), *N*-Hydroxysuccinimide (NHS) (0.276 g, 2.4 mmol) and 4-(*N,N*-Dimethylamino) butanoic acid (0.4 g, 2 mmol) were dissolved in dichloromethane (5 mL) and was added into a 100 mL round-bottom flask and stirred at 37°C for 24 h. The crude was extracted by DCM and washed three times with distilled water (20 mL). The product was purified by column chromatography (dichloromethane: methanol = 100:1 to 25:1, v/v). ^{1}H-NMR (300 MHz, $CDCl_3$), δ(ppm): 7.98 (s, 1H, NH).

Synthesis and characterization of BBZ

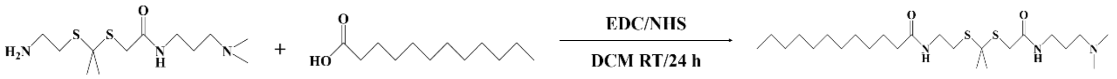

BBZ-4 (0.293 g, 0.1 mmol), EDC (0.459 g, 2.4 mmol), NHS (0.276 g, 2.4 mmol) and lauric acid (0.4 g, 2 mmol) were dissolved in DCM and was added into a 100 mL round-bottom flask and stirred at 37°C for 24 h. The crude was extracted by DCM and washed three times with distilled water (20 mL). The product was purified by column chromatography (dichloromethane: methanol = 100:1 to 10:1, v/v). ^{1}H-NMR (300 MHz, $CDCl_3$), δ(ppm): 0.9 (m, 3H, CH_3), 1.28-1.62 (m, 20H, CH_2), 2.21-2.30 (m, 6H, NCH_3), 2.80 (m, 2H, CH_2S), 3.49 (m, 6H, $NHCH_2$).

Synthesis and characterization of BBZN-1

BBZN-1

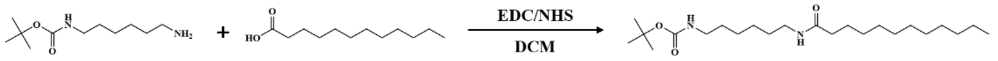

N-boc-1, 6-hexamediamine (0.648 g, 3 mmol), EDC (0.687 g, 3.6 mmol), NHS (0.414 g, 3.6 mmol) and lauric acid (0.6 g, 3 mmol) were dissolved in anhydrous dimethyl sulfoxide (15 mL) and was added into a 50 mL round-bottom flask and stirred at 40°C for 4 h. The solvent was precipitation with water and washed three times (20 mL). The product was purified by column chromatography (hexane: dichloromethane = 10:1 to 2:1, v/v). The white crystal was obtained with a yield of 95%. ^{1}H-NMR (300 MHz,

$CDCl_3$), δ(ppm): 0.9 (m, 3H, CH_3), 1.28-1.35 (m, 26H, CH_2), 1.42 (m, 9H, $C(CH_3)_3$), 2.18 (m, 2H, CH_2CONH), 3.13 (m, 2H, $NHCH_2$), 3.26 (m, 2H, $CONHCH_2$).

Synthesis and characterization of BBZN-2

BBZN-2

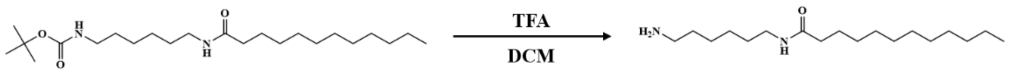

BBZN-1 (0.398 g, 1 mmol) and TFA (1 mL) were dissolved in anhydrous DCM and were added into a 25 mL round-bottom flask and stirred at room temperature for 1 h. The solvent was then washed with saturated sodium bicarbonate and extracted by DCM (20 mL) three times. A yellow oil was obtained with a yield of 65% and the product was without any other purification. ^{1}H-NMR (300 MHz, $CDCl_3$), δ(ppm): 0.9 (m, 3H, CH_3), 1.28-1.35 (m, 16H, CH_2), 1.64 (m, 2H, NH_2).

Synthesis and characterization of BBZN-3

BBZN-3

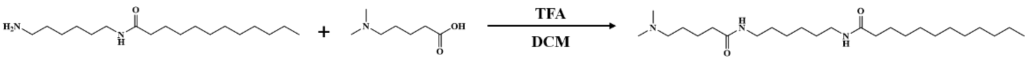

BBZN-2 (0.298 g, 1 mmol), EDC (0.229 g, 1.2 mmol), NHS (0.138 g, 1.2 mmol), and *N, N*-dimethylvalerate (0.145 g, 1 mmol) were dissolved in anhydrous dimethyl sulfoxide (20 mL) and was added into a 100 mL round-bottom flask and stirred at 40°C for 4 h. The solvent was precipitation with water and washed three times (20 mL). The product was purified by column chromatography (dichloromethane: methanol= 10:1 to 5:1, v/v). The white crystal was obtained with a yield of 47%. ^{1}H-NMR (300 MHz, $CDCl_3$), δ(ppm): 0.9 (m, 3H, CH_3), 1.28-1.35 (m, 26H, CH_2), 2.03 (m, 6H, $N(CH_3)_3$).

Preparation and characterization of lipid nanoparticles

Typically, the A/Y (lipid nanoparticles encapsulated AC73) was prepared by thin film dispersion and followed by ultrasonication[26]. Components of phospholipids, cholesterol, DSPE-mPEG$_{2000}$, and BBZ (w:w:w:w=30:5:5:15/30/60/120) were dissolved in DCM and the membrane materials' total weight was 15 mg. Then AC73 (0.3 mg) dissolved in methanol was added into the organic solvent mentioned above for the preparation of A/Y. After removing the solvent under the vacuum using a rotary

evaporator at 37°C for 10 min, a thin film was observed and the distilled water was added for hydrating immediately. The hydration process takes place at 37 °C and lasts for 30 minutes. The A/Y lipid nanoparticles (lipid NPs) were obtained by ultrasonication for 3 min. The UA/Y (encapsulated AC73 and siUSP-1) and MUA/Y (encapsulated AC73, siUSP-1 and MFG-E8) were prepared by charge interaction between A/Y and siUSP-1 or MFG-E8. siUSP-1 (0.1 OD) and MFG-E8 (0.02 mg) were dissolved in DEPC-treated water (DEPC water). Finial lipid NPs were prepared by adding equal volumes of A/Y and DEPC water with gene and protein to attain different w/w ratios stirred for 30 s and incubated at room temperature. Then, the obtained liposomes were centrifuged at 10000 rpm for 5 min, purified using ultrafiltration filters (MWCO = 10 kDa), and stored at 4°C before further use. The C6/Y NPs preparation method was as same as mentioned above. Size distribution and zeta potential of A/Y and MUA/Y were measured by a ZetaPlus particle size analyzer (Brookhaven Instruments, USA). The Transmission electron microscope (TEM, JEM-200CX, JEOL, Japan) was also used to visualize the appearance of the NPs. UV-vis was used to determine AC73 and MUA/Y NPs between 200 to 800 nm. Also, the drug loading efficiency (DLE) and drug encapsulation efficiency (EE) were measured by UV-vis method. siRNA condensing ability of lipid NPs as measured by agarose gel electrophoresis at 100 V for 15 min. The ability of NPs to resist the degradation of RNase A was investigated by incubating with RNase at 37°C for 3 h. Then add 5 μL 2% sodium dodecyl sulfate (SDS) to the sample to replace siRNA. siRNA integrity was determined by gel electrophoresis as mentioned above.

The stability of the lipid NPs colloid was incubated in PBS, saline, and rat serum at 37°C for 48 h, and then the hydrodynamic diameter after different incubation times.

To verify the MFG-E8 could be adsorbed on MUA/Y NPs through electrostatic interaction. We modified RhB on MFG-E8 and incubated FITC in Y lipid nanoparticles respectively and obtained RhB-MFG-E8/FITC-Y using the method which was widely used. The Fluorescence Resonance Energy Transfer (FRET) technique provides the proximal information of the two fluorescent molecules within a 1-10 nm range as the energy transfer from a donor to an acceptor. The FITC and RhB pair acted as the donor

and receptor of fluorescence excitation. The detection wavelength was 488 nm and the emission wavelength was between 500 nm to 700 nm.

EE and DLE of AC73 were determined by the UV method. Briefly, free AC73 was totally removed by ultrafiltration centrifugation and then methyl alcohol was used to destroy the NPs structure. AC73 entrapped in nanoparticles and total AC73 were detected by ultraviolet spectrum. EE and DLE were calculated referring to the following formulas:

$$EE(\%)=\frac{W_{\text{entrapped AC73}}}{W_{\text{total AC73}}}\times 100\%$$

$$DLE(\%)=\frac{W_{\text{entrapped AC73}}}{W_{\text{materials and AC73}}}\times 100\%$$

Drug release and ROS-sensitive detection

The *in vitro* AC73 release from MUA/Y liposomes was analyzed by the dialysis method. 1 mL MUA/Y lipid nanoparticles were placed in a tightly sealed dialysis bag (MWCO 3500-5000). Immediately, 30 mL PBS (pH 5.0, pH 7.4 with 0.1% Tween 80 and with or without H_2O_2). The dialysis bag was then put under 37°C environment and mechanical shaking at 100 rpm. After the desired time points, accurately remove 0.5 mL PBS from each group and timely supplement pre-warmed fresh medium. AC73 quality was measured as the method mentioned above.

The detection of ROS-sensitive can also use the DCFH-DA method *in vitro*. Firstly, HSCs were incubated in a 6-well microplate. When the cell confluence reached 70-80%, PBS and MUA/Y were added and then incubated for 12 h. Subsequently, the DCFH-DA solution replaced the medium. The ROS degradation was observed under a fluorescence microscope.

Collagen degradation *in vitro* mediated by MFG-E8

The mixture of matrigel and serum-free medium was vortex under the ratio of 1:3. Then transwell chambers were incubated at 37°C for 2 h until the matrigel solidified after adding 40 μL above the mixture to each chamber. MFG-E8, murine macrophage cell line (RAW264.7) cells, MFG-E8 with RAW264.7, M/Y with RAW264.7, and MU/Y

with RAW264.7 were added into each chamber and then matched with the core which pre-laid HSCs (8 ×104 well^{-1}). The fluorescence intensity in HSC was observed by fluorescence microscope to evaluate the collagen degradation efficiency.

In order to visually observe collagen degradation, FITC-labeled collagen was pre-laid in a 6-well microplate (8 ×10^4 well^{-1}), and then the above groups were added to observe the fluorescence intensity in RAW264.7 cells via fluorescence microscope to evaluate the degree of collagen degradation.

Cellular uptake, viability, apoptosis, and intracellular trafficking

HSCs were pre-seeded in a 6-well plate and then added different formulations (Free C6, C6/Y, and MUC6/Y) at different time intervals (2 h and 4 h). Then the mean fluorescence intensity (MFI) was analyzed by flow cytometry and laser confocal microscope.

HSC, RAW264.7, and L02 were cultured in DMEM for cell studies. Cell viability assay was evaluated by the MTT method. Specifically, a seriously increasing concentration of different formulations (Free AC73, A/Y, and MUA/Y) were cocultured with HSCs and incubated for 24 h. After that, the cell viability was calculated by the cells only cultured under PBS.

For the apoptosis study, pre-set concentrations of different formulations were added into the medium and collected cells through flow cytometry.

For endocytosis pathways, chlorpromazine (an inhibitor of clathrin-mediated endocytosis), amiloride (an inhibitor of micropinocytosis), and methyl-β-cyclodextrin (MβCD) (an inhibitor of caveolae-mediated endocytosis) were first pre-incubated with HSC cells for 1 h, and another group was cultured in 4°C. Then C6/Y was added into the supplement medium immediately and incubated for another 2 h. Cells were collected and detected via flow cytometry to qualify the C6-positive cells for the calculation of the suppression of cell uptake.

Lysosome escape assay

The HSC cells were cultured into the 6-well microplate (8 ×10^4 well^{-1}), and incubated with Free siFAM, siFAM/ Y, and M/siFAM/ Y (2 μg siFAM well^{-1}) for 3 h and 6 h.

Subsequently, the lysosomes of cells were stained with Lyso-Tracker Red DND-99 kit (10 μg mL^{-1}, 1 h).

In vitro siRNA transfection and gene silencing

The *in vitro* siRNA transfection ability was measured by 4T1-Luc cells (Luc stably stable express cells), siRNA concentration in each formulation was 2 μg mL^{-1}. Different formulations (Free siLuc, siLuc/$lipo_{2000}$, siLuc/Y, and M/siLuc/Y) with 10% FBS were added for 4 h and incubated in a dark environment for another 44 h. Luc expression in 4T1 cells was detected by fluorescence microscope.

In order to verify the effect of different concentrations of serum on gene silencing efficiency. 20% FBS and 30% FBS were selected to replace the above 10% FBS supplement medium, respectively. The relative luciferase expression rate was calculated by the control group.

In vitro anti-hepatic fibrosis study

HSCs were co-cultured with TGF-β (10 ng mL^{-1}). After cells reached 70–80% confluence and then add different formulations (PBS, Y, free AC73, AC/Y, and MUA/Y). Cells were homogenized using ice-cold RIPA buffer and analyzed the expression of α-SMA, collagen I, CD147, fibronectin, USP-1, and Sp-1.

Immunofluorescence staining

The distribution of Free AC73 and MUA/Y in mice liver was investigated by immunofluorescence staining. DiI solution and MUDiR/Y containing equal DiI amount (DiI concentration: 200 ug kg^{-1}) were intravenously injected into fibrosis mice, respectively. The fibrosis mice model was constructed as the method has been reported. After 4 h, mice were sacrificed and liver tissue was used to make frozen sections (8 mm) by a cryostat microtome (Leica, CM1960, Germany) kept at -80°C.

In vivo biodistribution

DiR was encapsulated in the MUDiR/Y to localize and monitor the nanoparticle biodistribution. IVIS Spectrum CT *in vivo* imaging system (Caliper Life Sciences Inc., Mountain View, CA, USA) was used to detect the fluorescence intensity of mice.

Briefly, the fibrosis mice model was constructed as the method has been reported. Free DiR and MUDiR/Y were intravenously injected at a dose of 200 μg kg^{-1}. Post-injection *in vivo* images were obtained at 3 h, 6 h, 9 h, 12 h, 24 h, and 48 h using the IVIS Spectrum. And then the fluorescence intensity of Free DiR and MUDiR/Y was measured at 12 h in all the major organs.

Animal study

Six weeks old male ICR mice were purchased from Nanjing Cavans Biotechnology (Co, Ltd, China). All animal experiments followed the protocol approved by China Pharmaceutical University. To obtain a fibrotic mice model, ICR mice were injected intraperitoneally with 5% CCl_4 (5 mL kg^{-1}) dissolved in olive oil 3 times a week for 11 weeks. The body weight was measured accurately after each injection. In the last two weeks, each different formulation (PBS, free AC73, free MFG-E8, A/Y, U/Y, MUA/Y) was intravenously injected via the tail vein (the quality of AC73, MFG-E8, and siUSP1 in each group was 3mg kg^{-1}, 20 μg kg^{-1}, and 1.5 mg kg^{-1} per day. After the last time treatment, mice were sacrificed. Mice weight and liver weight were measured accurately. AST, ALT, ALP, BUN, and CR levels were measured using colorimetric kits. Hydroxyproline (Hyp) was tested with a hydroxyproline assay kit (Nanjing Jiancheng Bioengineering Institute, Nanjing, China). Hematoxylin and eosin (H&E), Masson, and Sirius red staining of liver sections were used to carry out a histological evaluation of liver damage and examine the anti-fibrotic effect of each formulation. Furthermore, the serum levels of IL-6, IL-1β, TNF-α, and TGF-β were tested by ELISA assay kit (Nanjing Jiancheng Bioengineering Institute, Nanjing, China).

The mouse MCD model was obtained by feeding mice a methionine-choline deficient (MCD) diet. After 6 weeks of feeding, the mice were sacrificed and the liver was collected. The detection indexes were the same as those of the CCl_4 model. The expression of α-SMA, collagen I, fibronectin, CD147, USP1 and Sp1 were analyzed in the liver according to a standard procedure and their bands were analyzed using Image J. The expression of collagen I was determined by Sirius red staining and α-SMA, CD147, iNOS, and CD206 were determined by immunofluorescence according to a

standard procedure and their positive areas were analyzed using Image J.

Hemolysis assay

Briefly, mouse blood (500 μL) was mixed with equal PBS and obtained red blood cells after repeat centrifugation (3000 rpm, 5 min). 800 μL of MUA/Y lipid nanoparticles with different concentrations were added into 200 μL red blood cell suspension (2% in PBS), and PBS with or without 2% Triton X-100 were respectively regarded as positive and negative controls. After incubation at 37 ◦C for 3 h, the absorbance of the sample supernatant was measured at 540 nm. Pictures were taken and the percentage of hemolysis was calculated simultaneously.

Statistical analysis

All the details about sample size, data presentation, statistical analysis, and significant differences are provided in the figure captions. Experiments were conducted three times at least and all data are presented as mean ± S.D. GraphPad (version 9.0) was applied for graphing. Unpaired two-tailed student's t-test was used for two-group comparison and one-way analysis of variance (ANOVA) to detect the significant differences between multiple groups. $p > 0.05$ represented no significant difference (n.s.), *$p < 0.05$ represented a significant difference, and **$p < 0.01$ and ***$p < 0.001$ represented a highly significant difference. Image J software was applied for quantitative analysis.

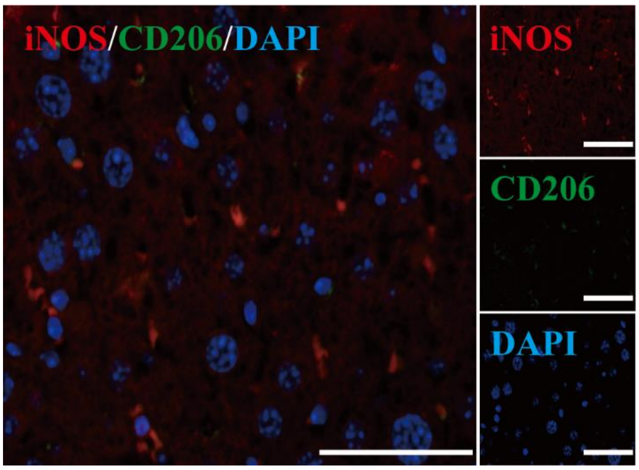

Figure S1. The abundance of M1 macrophages (labeled with Alexa Flour 647) and M2 macrophages (labeled with FITC) in fibrotic liver. Scale bar = 200 μm.

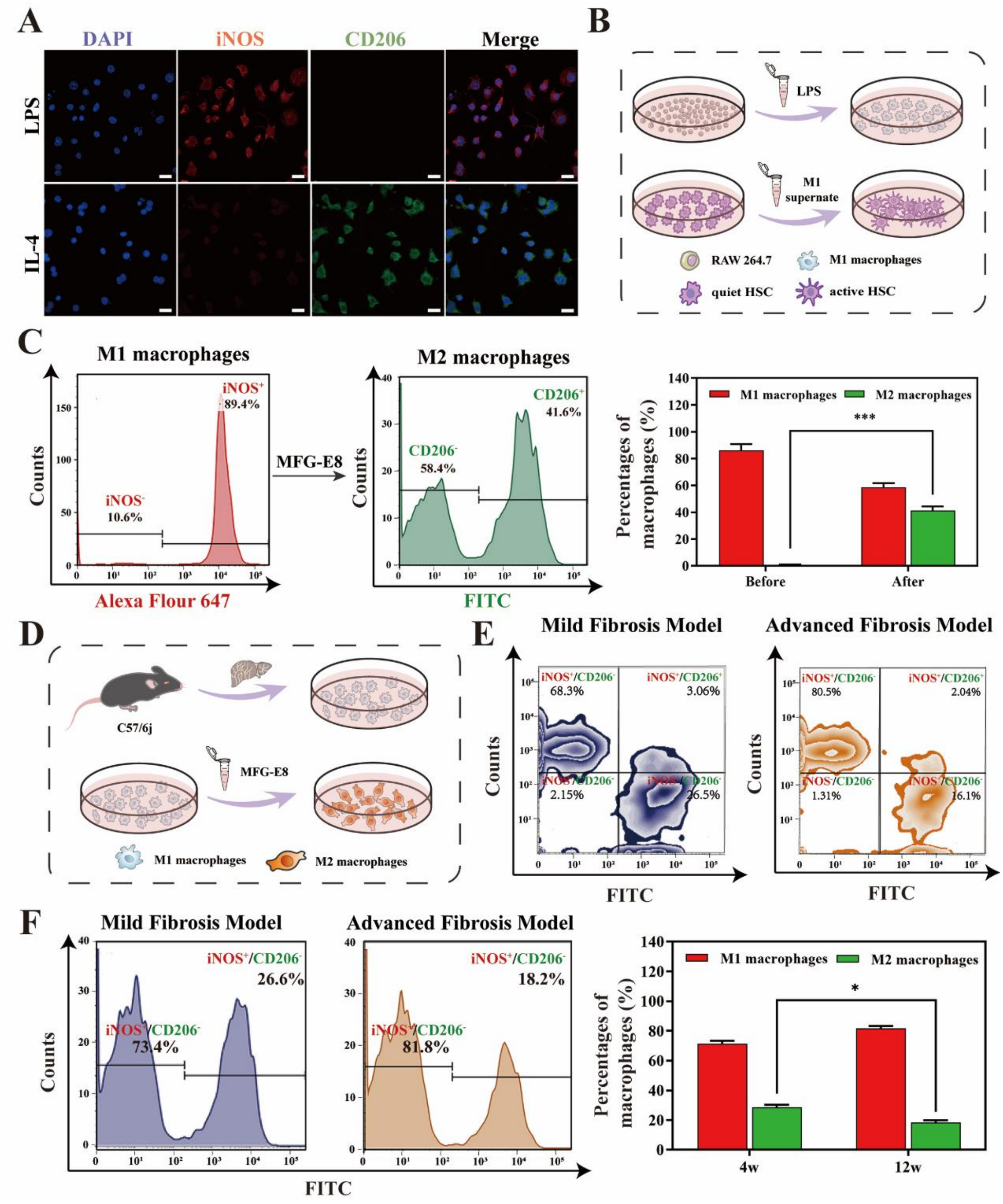

Figure S2. Macrophages influence the progression of LF. A) Laser confocal microscope (CLSM) images of immunofluorescence staining of iNOS and CD206. Scale bar = 20 μm. B) The process of macrophage supernatants activated HSCs. C) Detection of iNOS and CD206 expression in macrophages after induced by MFG-E8 using flow cytometry, (n = 3). D) The process of macrophage phenotype reversion by MFG-E8 from fibrosis mice. E) Detection of iNOS and CD206 expression in macrophages separated from fibrosis after induced by MFG-E8 using flow cytometry. F) Statistics analysis of M1 and M2 macrophage ratio in fibrosis mice (n = 3). Data were represented as mean ±SD, *p < 0.05, ***p < 0.001.

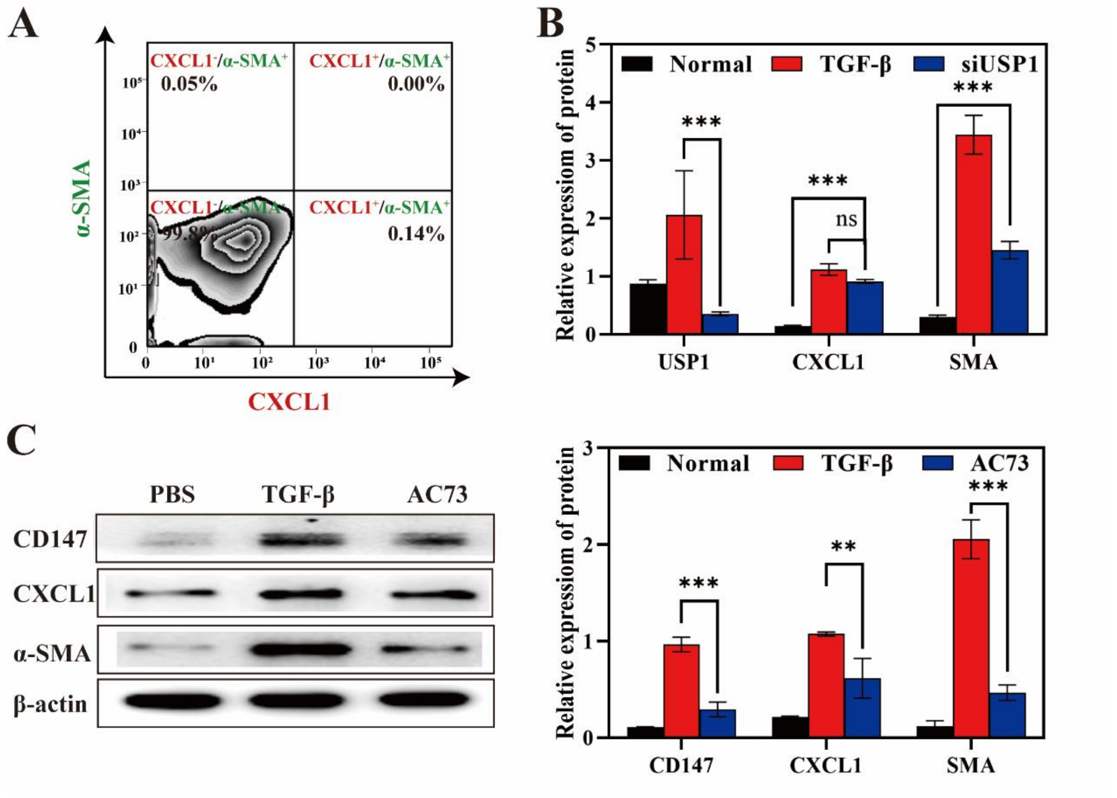

Figure S3. A) The expression of CXCL1 and α-SMA in quiescence HSC was detected by flow cytometry. B) Statistics analysis of the USP1, CXCL1, and α-SMA expression in HSC after undergoing different treatments. The expression of USP1, CXCL1, and α-SMA were normalized to β-actin, (n = 3). C) Western blot analysis of CD147, CXCL1, and α-SMA in HSCs after undergoing different treatments. The expression of CD147, CXCL1, and α-SMA were normalized to β-actin, (n = 3). Data were represented as mean ±SD, ns means no significant difference, *p < 0.05, **p < 0.01, ***p < 0.001.

BBZ-1

BBZ-2

BBZ-3

BBZ-4

BBZ

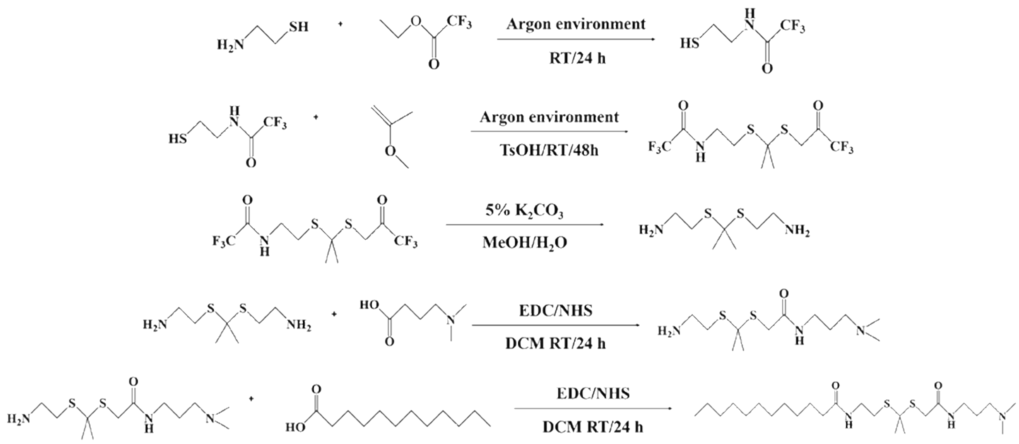

Figure S4. The synthesis routine of BBZ.

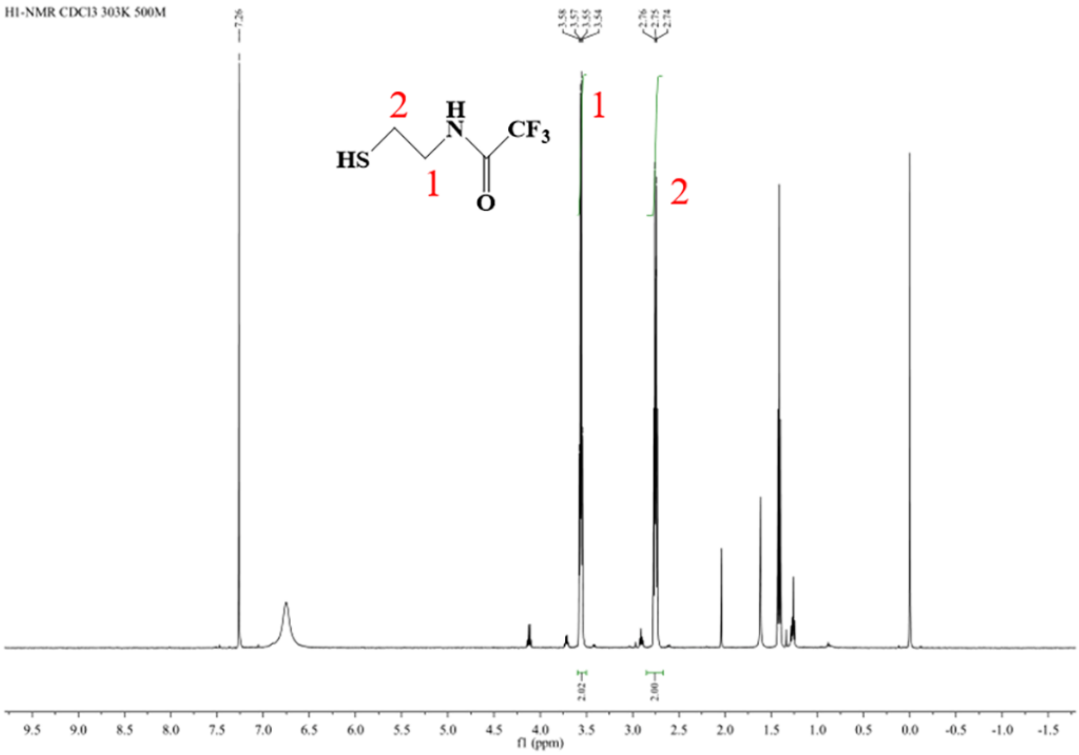

Figure S5. ^{1}H-NMR spectrum of BBZ-1 in $CDCl_3$.

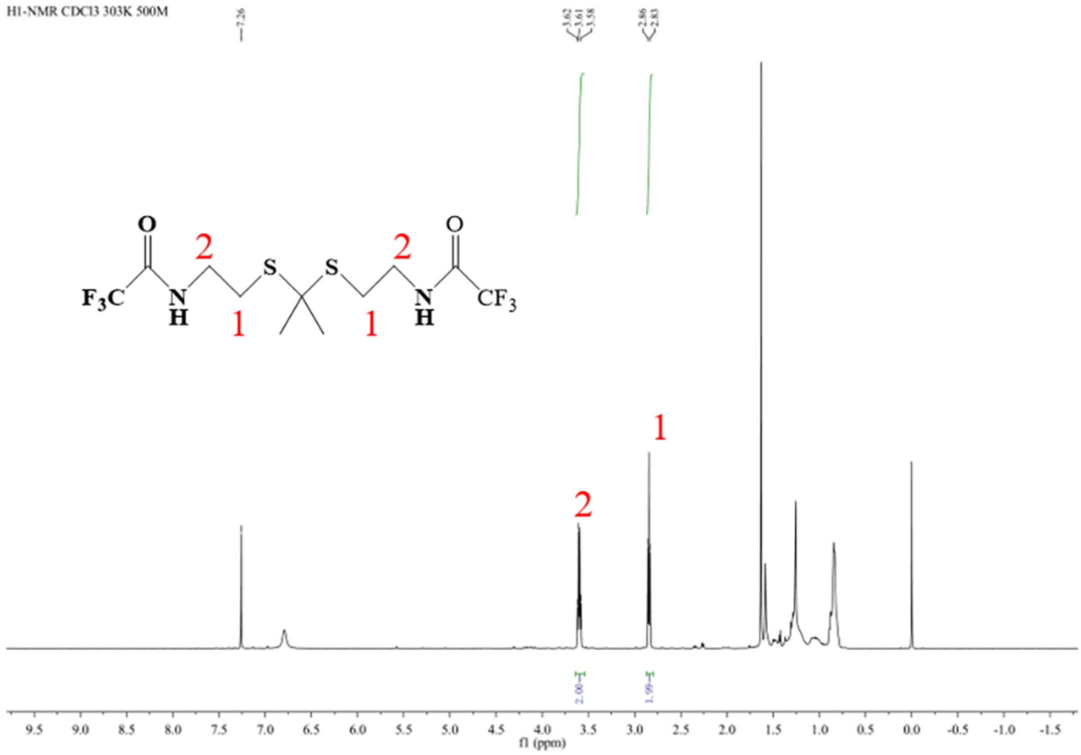

Figure S6. ^{1}H-NMR spectrum of BBZ-2 in $CDCl_3$.

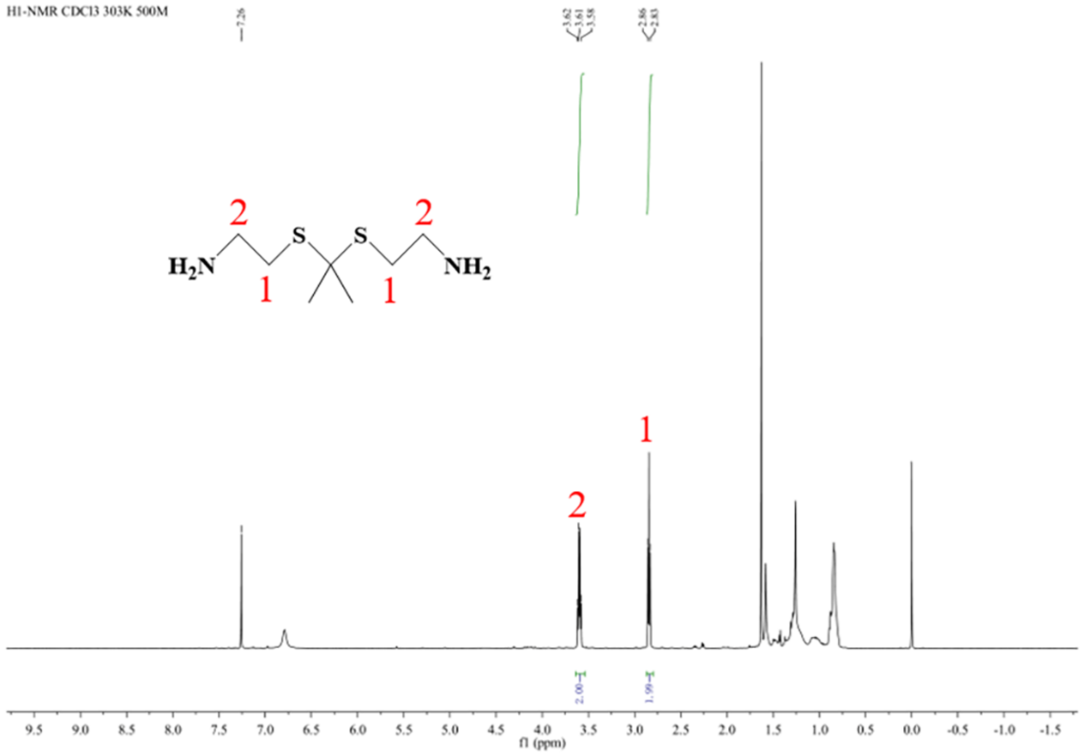

Figure S7. ^{1}H-NMR spectrum of BBZ-3 in $CDCl_3$.

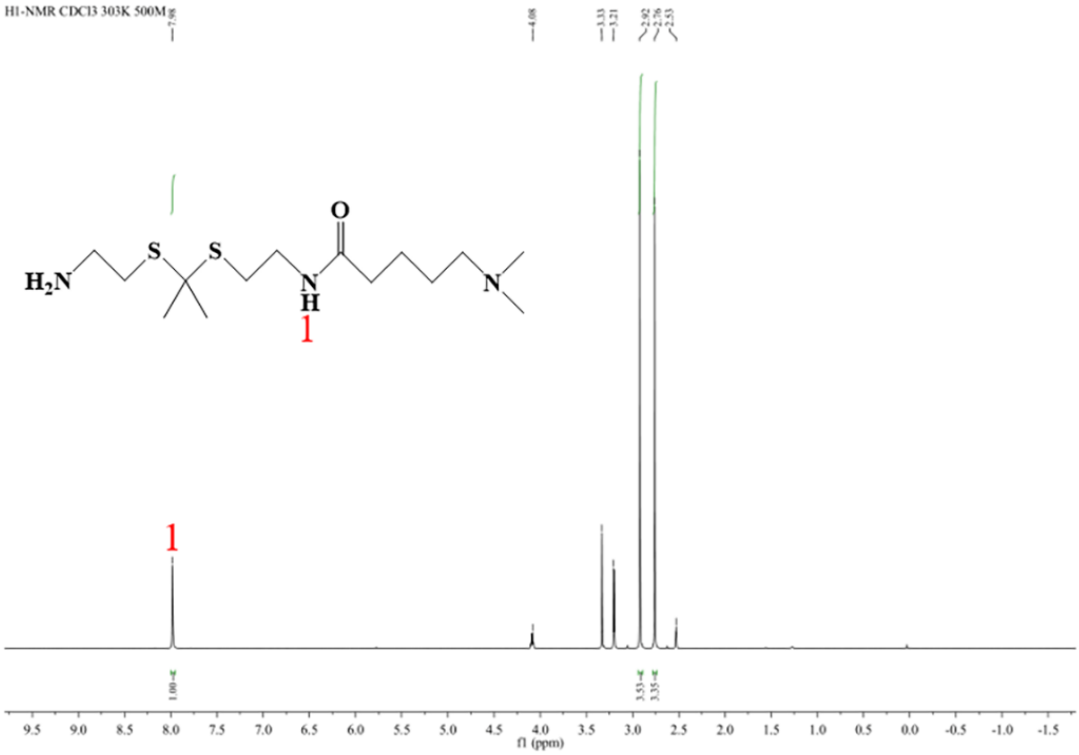

Figure S8. ^{1}H-NMR spectrum of BBZ-4 in $CDCl_3$.

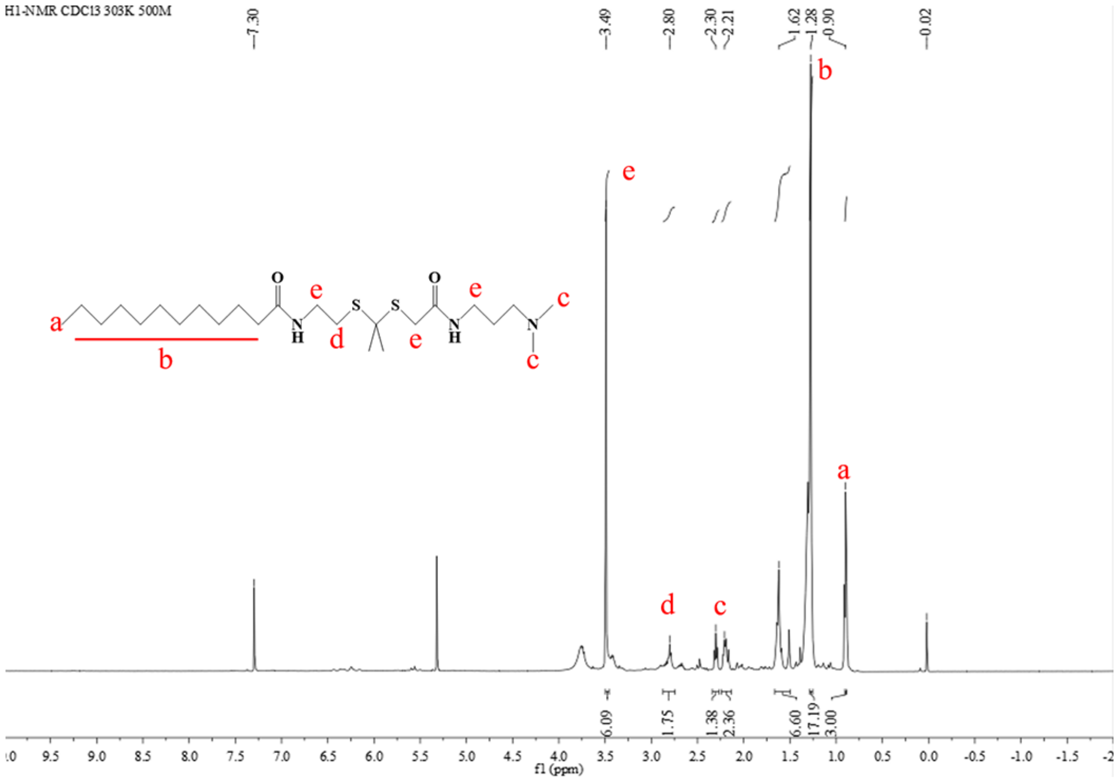

Figure S9. ^{1}H-NMR spectrum of BBZ in $CDCl_3$.

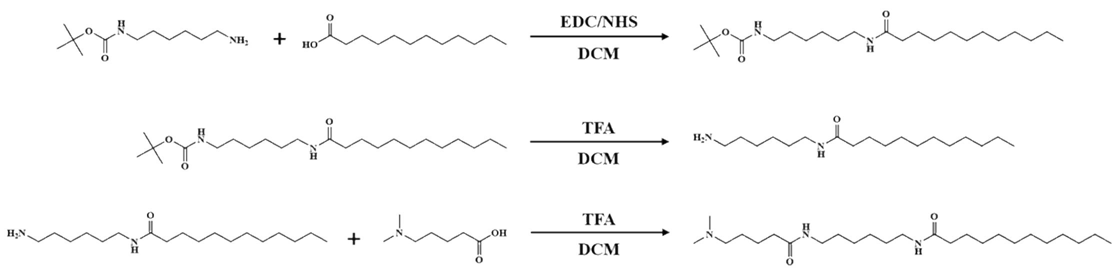

Figure S10. The synthesis routine of BBZN1-BBZN3.

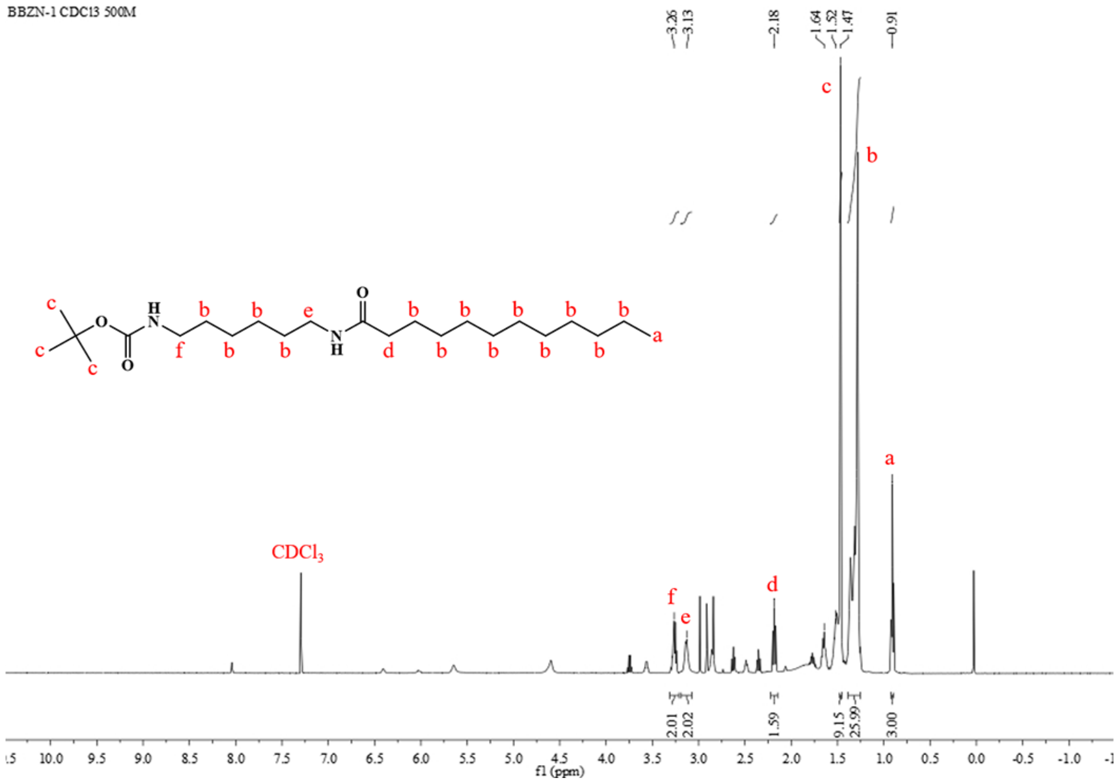

Figure S11. ^{1}H-NMR spectrum of BBZN-1 in $CDCl_3$.

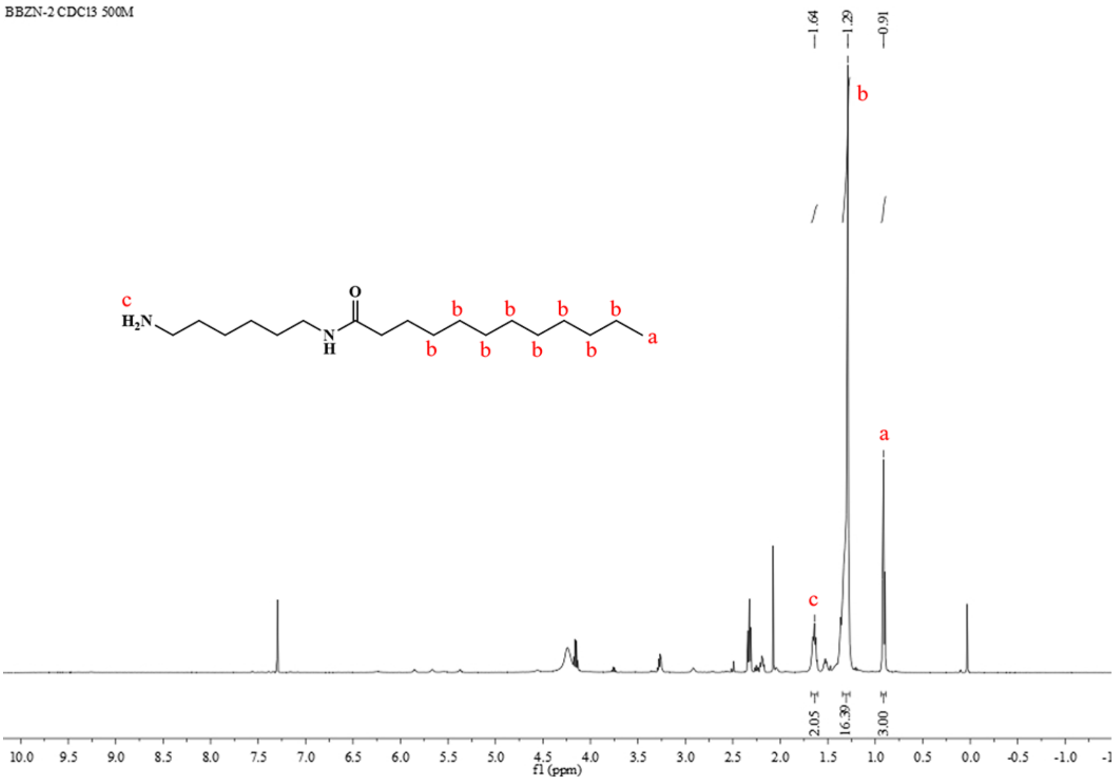

Figure S12. ^{1}H-NMR spectrum of BBZN-2 in $CDCl_3$.

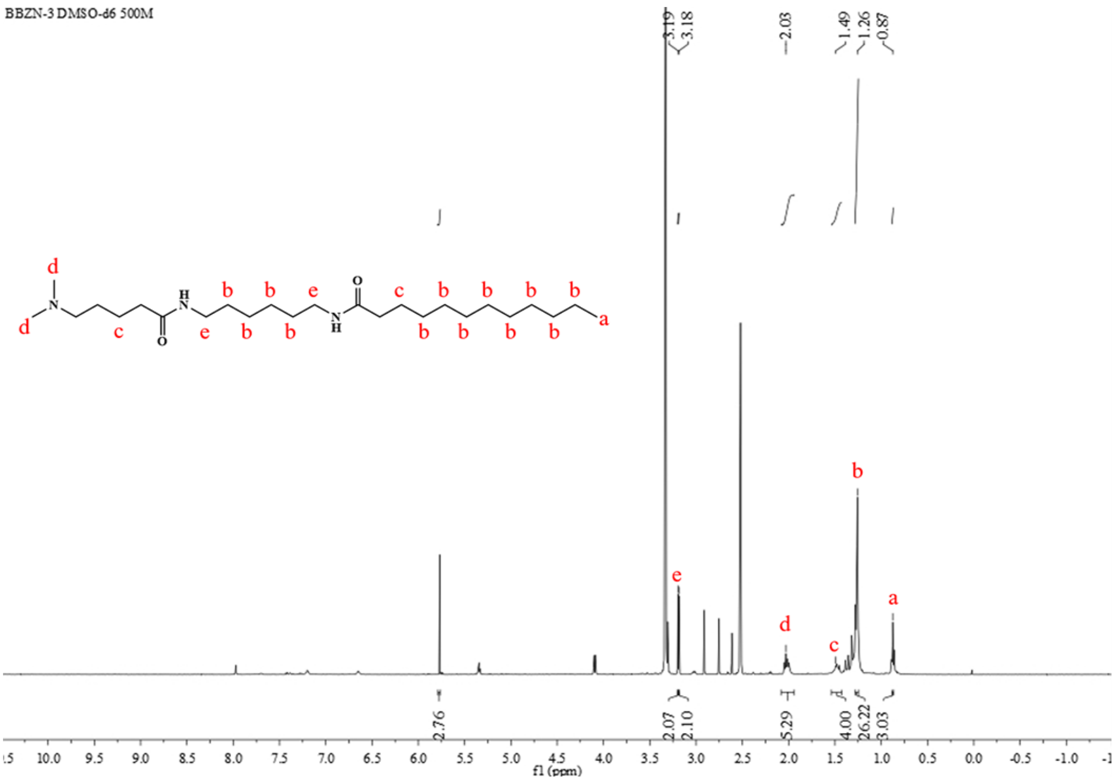

Figure S13. ^{1}H-NMR spectrum of BBZN-3 in DMSO-d_6.

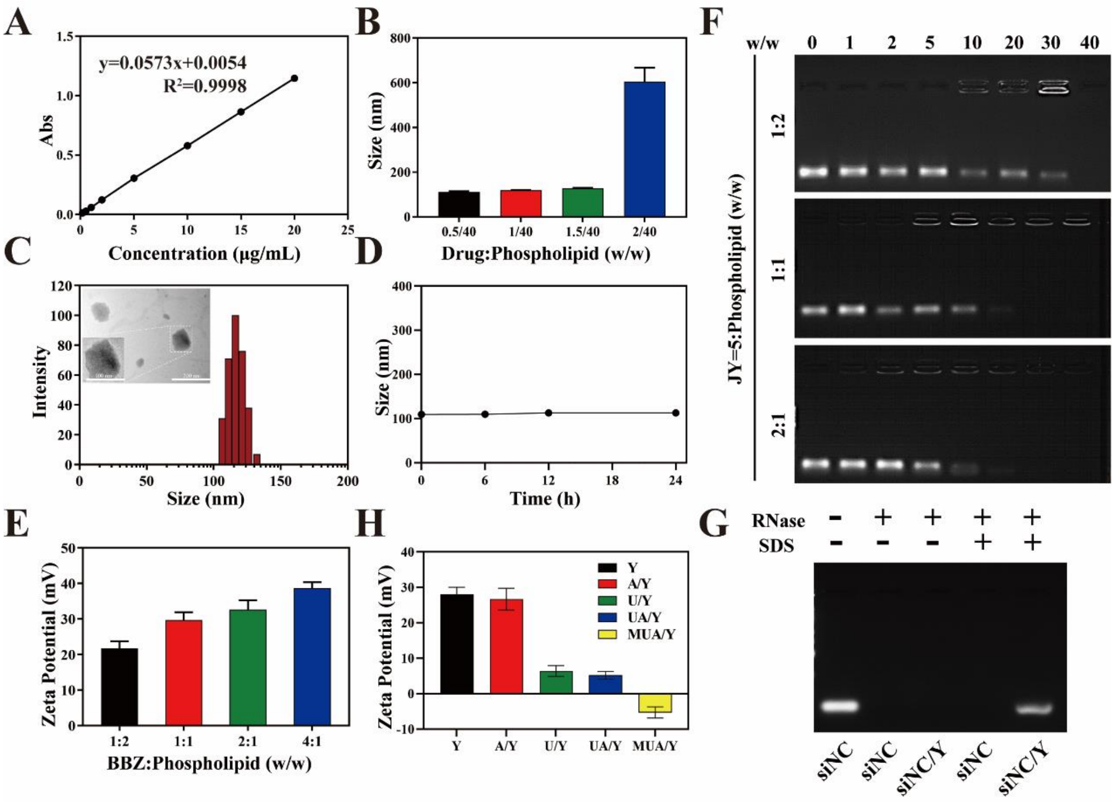

Figure S14. A) The standard curve of AC73 was detected under 365 nm using an ultraviolet spectrophotometer. B) Size distribution of A/Y lipid nanoparticles under different drug/phospholipid ratios (n = 3). C) Size distribution and TEM result of A/Y. TEM Scale bar = 200 nm. D) *In vitro* colloidal stability of A/Y in PBS for 24 h (n = 3). E) Zeta potential of different lipid nanoparticles under different BBZ/phospholipid ratios (n = 3). F) The siRNA binding ability of different BBZ/phospholipid ratios. G) The stability of siNC/Y lipid nanoparticles. H) Zeta potential of different formulations (n = 3). Data were presented as mean ± SD.

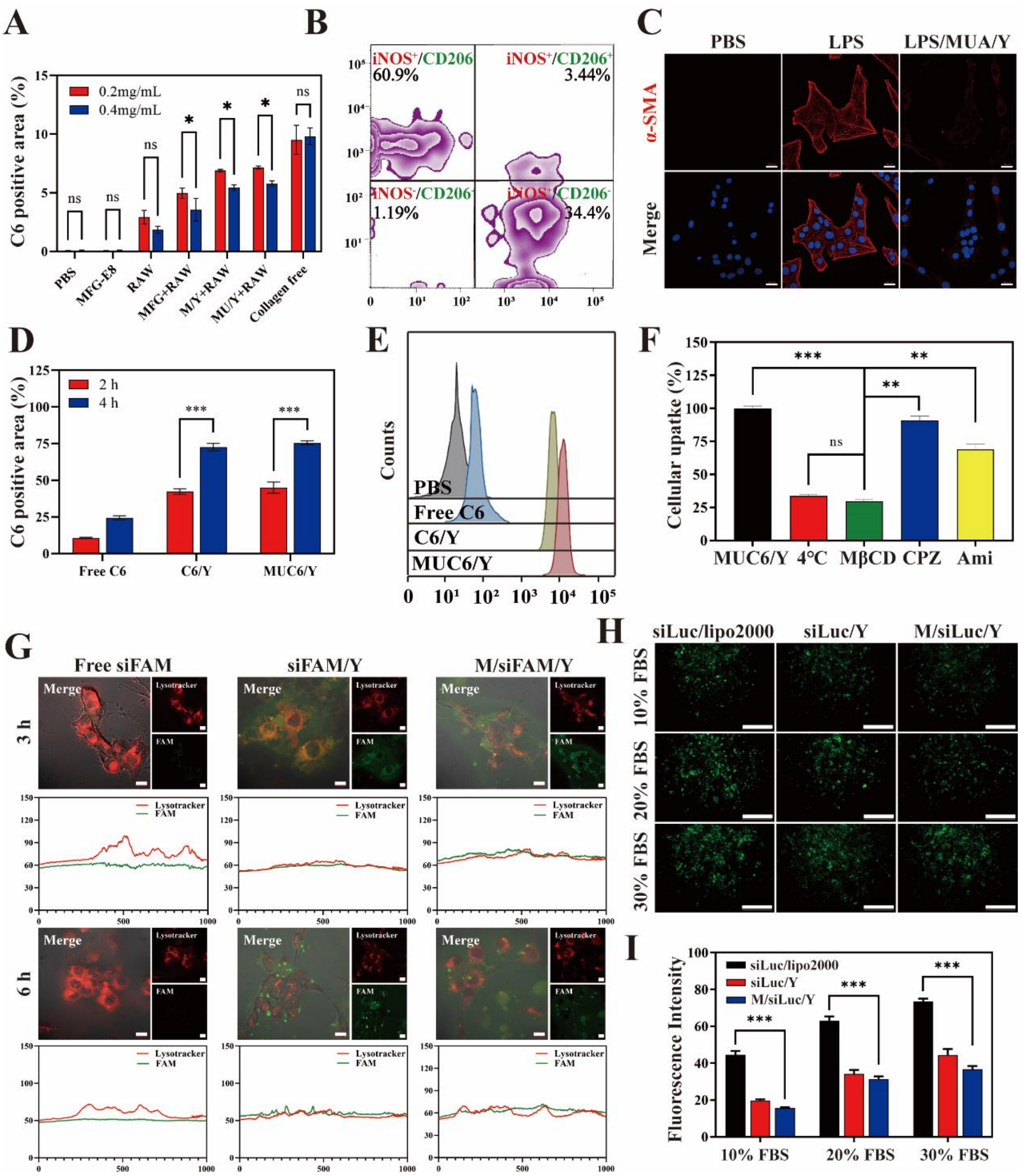

Figure S15. A) Semiquantitative analysis of C6 fluorescence intensity in HSCs after undergoing different formulations (MFG, RAW, MFG+ RAW, M/Y+ RAW, and MU/Y+ RAW) (n = 3). B) Detection of iNOS and CD206 expression by flow cytometry after incubation with MUA/Y lipid nanoparticles. C) CLSM images of immunofluorescence staining of α-SMA of HSC after activation by M1 macrophage supernatant preincubated with MUA/Y lipid nanoparticles. Scale bar = 20 μm. D) Semiquantitative analysis of C6 cellular uptake in HSCs in CLSM image (n = 3). E) Cellular uptake of MUA/Y lipid nanoparticles detected by flow cytometry. F) The uptake mechanism study (n = 3). G) Endosomal escape of Free siFAM, siFAM/Y, and M/siFAM/Y lipid nanoparticles after incubation for 3 and 6 h, observed by CLSM, where endo/lysosomes were stained with LysoTracker Red (red). Scale bar = 20 μm. H)Transfection efficiency of siLuc-loaded lipid nanoparticles on B16F10-Luc cells in the absence of 10%, 20%, and 30% FBS. Scale bars = 100 μm. I) Statistics analysis of the fluorescence area of B16F10-Luc cells (n = 3). Data were presented as mean ± SD, *p < 0.05, **p < 0.01, ***p < 0.001, ns means no significant difference.

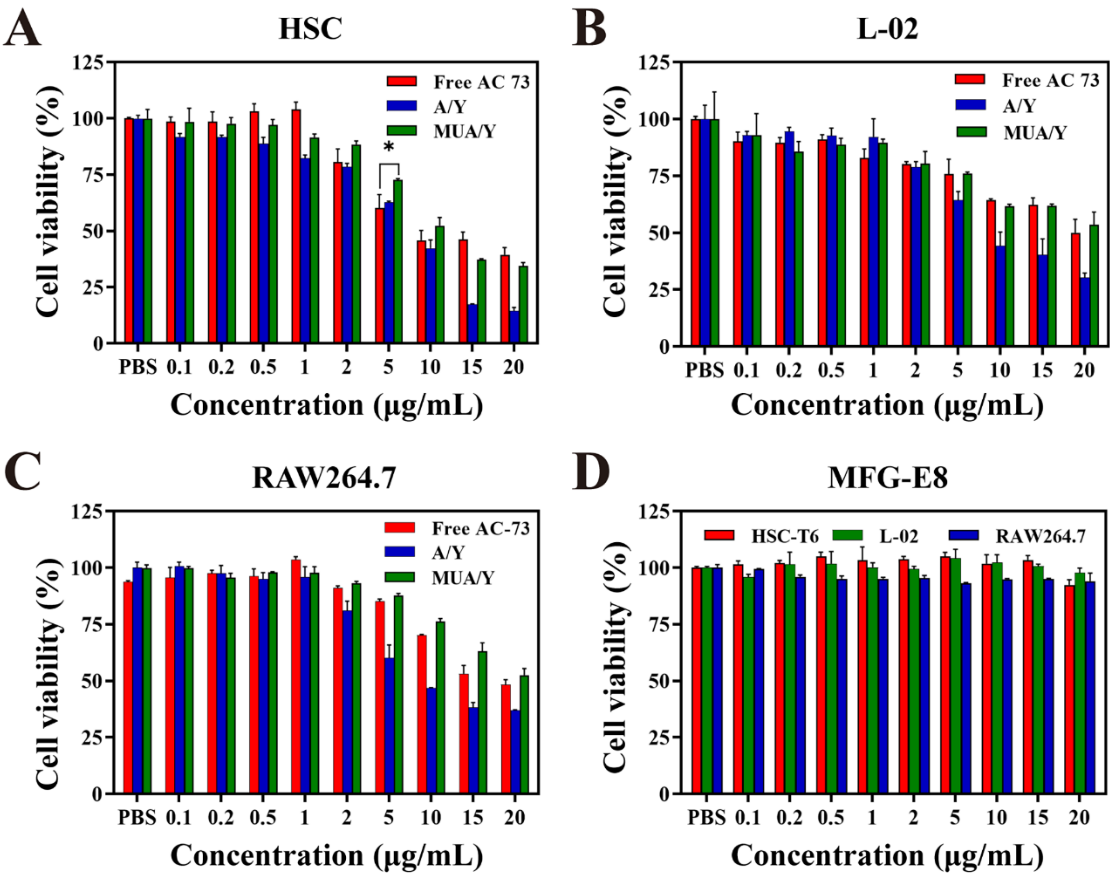

Figure S16. Cytotoxicity of MUA/Y lipid nanoparticles. A-C) Cell viability of the MUA/Y in HSC-T6, L02, and RAW264.7 cells (n = 3). D) Cell viability of the MFG-E8 in HSC-T6, L02, and RAW264.7 cells (n = 3). Data represented mean ±SD, *$p < 0.05$.

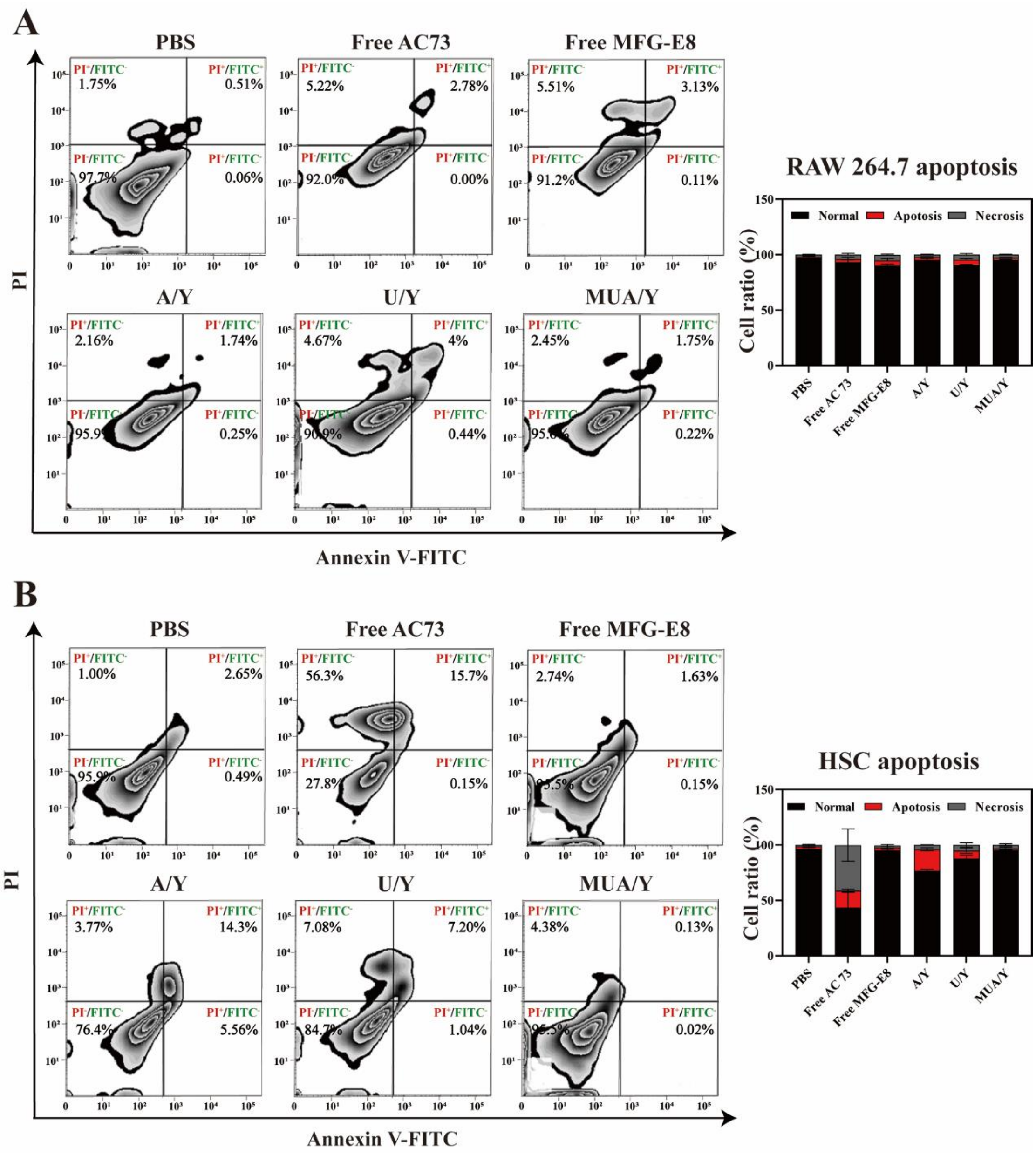

Figure S17. Effect of different formulations on apoptosis in A) RAW264.7 cells and B) HSC-T6 cells. Data represented mean ±SD, n = 3.

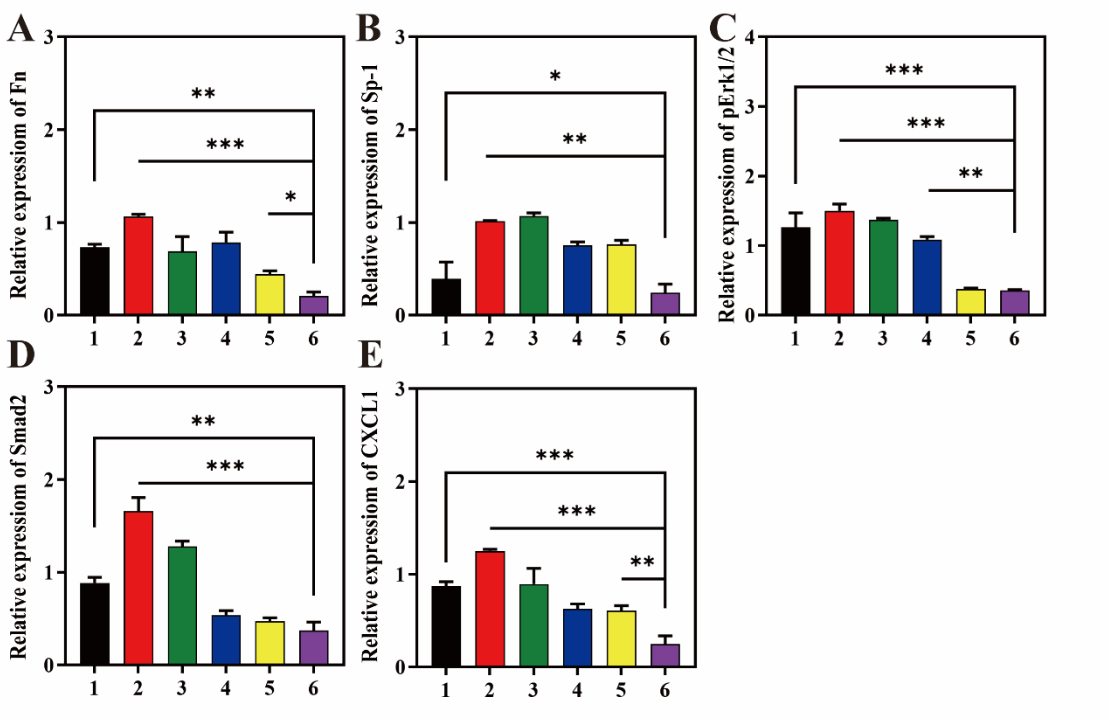

Figure S18. Statistics analysis of fibronectin (Fn), Sp1, Erk1/2, Smad2, and CXCL1 expression in HSCs undergoing different treatments. The expression of fibronectin, Sp1, Erk1/2, Smad2, and CXCL1 were normalized to β-actin. Data were represented as mean ±SD, n = 3. ns means no significant difference, *p < 0.05, **p < 0.01, ***p < 0.001.

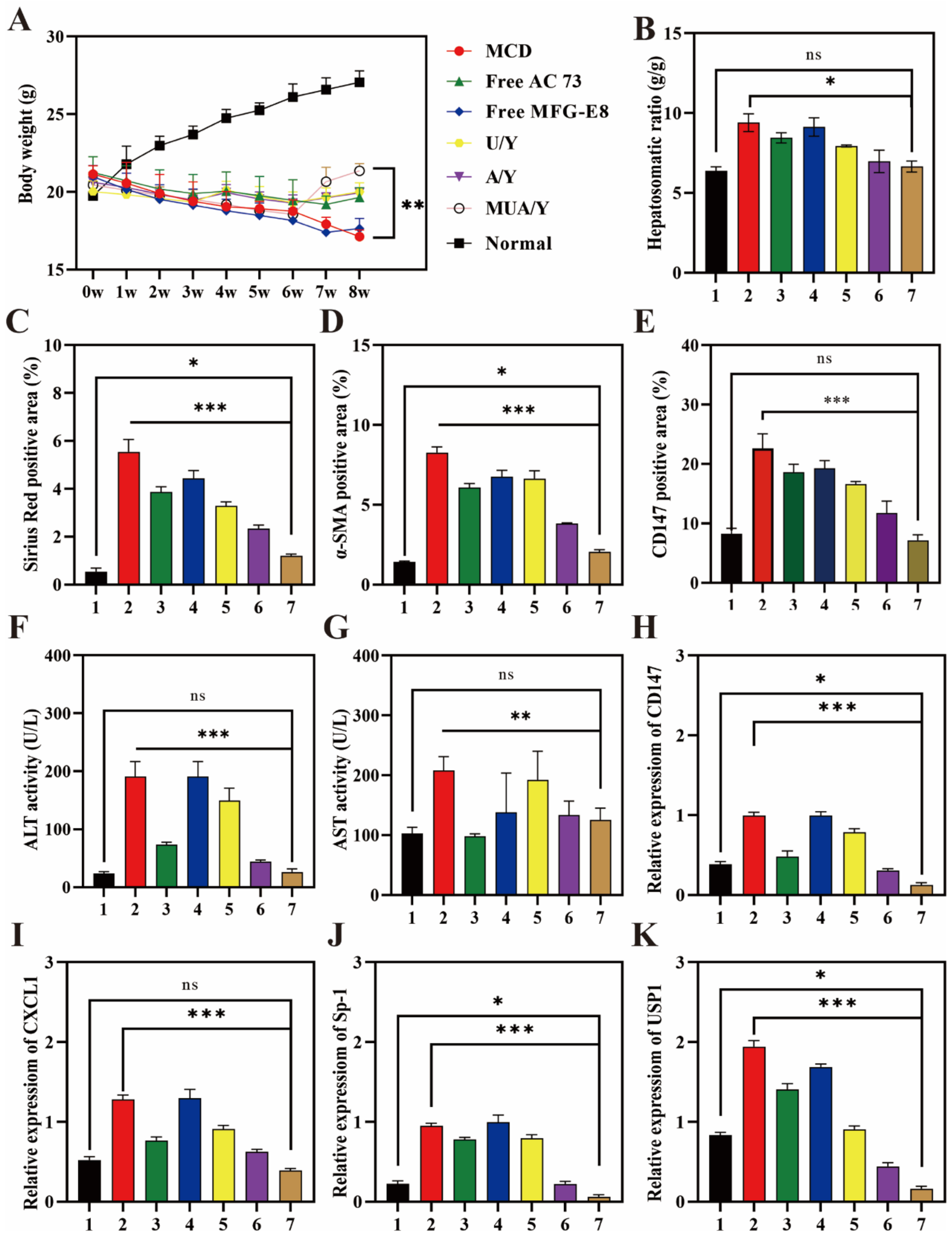

Figure S19. A) Body weight changes of different groups in the experiment of fibrosis remission, (n = 5). B) Hepatosomatic ratio of different groups, (n = 5). C) Semiquantitative analysis of the Sirius-red-positive area, (n = 3). D) Semiquantitative analysis of the α-SMA-positive area, (n = 3). E) Semiquantitative analysis of the CD147-positive area, (n = 3). F) Serum ALT activity, (n = 3). G) Serum AST activity, (n = 3). H-K) Relative expression levels of CD147, CXCL1, Sp1, and USP1. CD147, CXCL1, Sp1, and USP1 were normalized to β-actin, (n = 3). 1 means normal mice. 2 means fibrotic mice; 3 means free AC73 group; 4 means free MFG-E8 group; 5 means U/Y group; 6 means A/Y group; 7 means MUA/Y group; C-K) Data were represented as mean ±SD, *p < 0.05, **p < 0.01, ***p < 0.001.; NS: no significant difference.

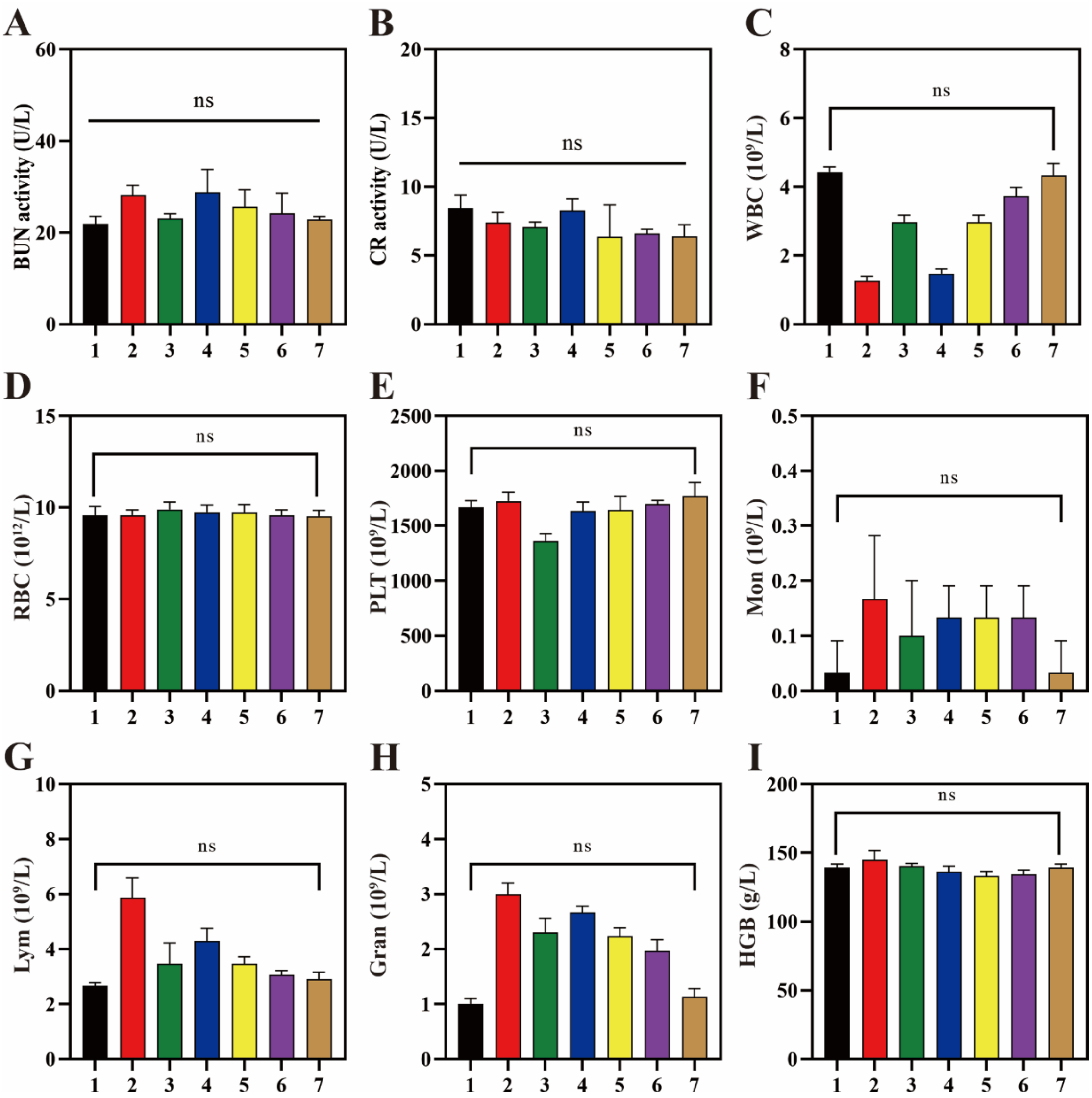

Figure S20. A) Serum BUN activity, (n = 3). B) Serum CR activity, (n = 3). C-I) The levels of physiological markers in different treatment groups, (n = 3). 1 means normal mice. 2 means fibrotic mice treated with PBS; 3 means fibrotic mice treated with free AC73; 4 means fibrotic mice treated with free MFG-E8; 5 means fibrotic mice treated with U/Y; 6 means mice treated with A/Y; 7 means mice treated with MUA/Y. Data were represented as mean ±SD, ns: no significant difference.

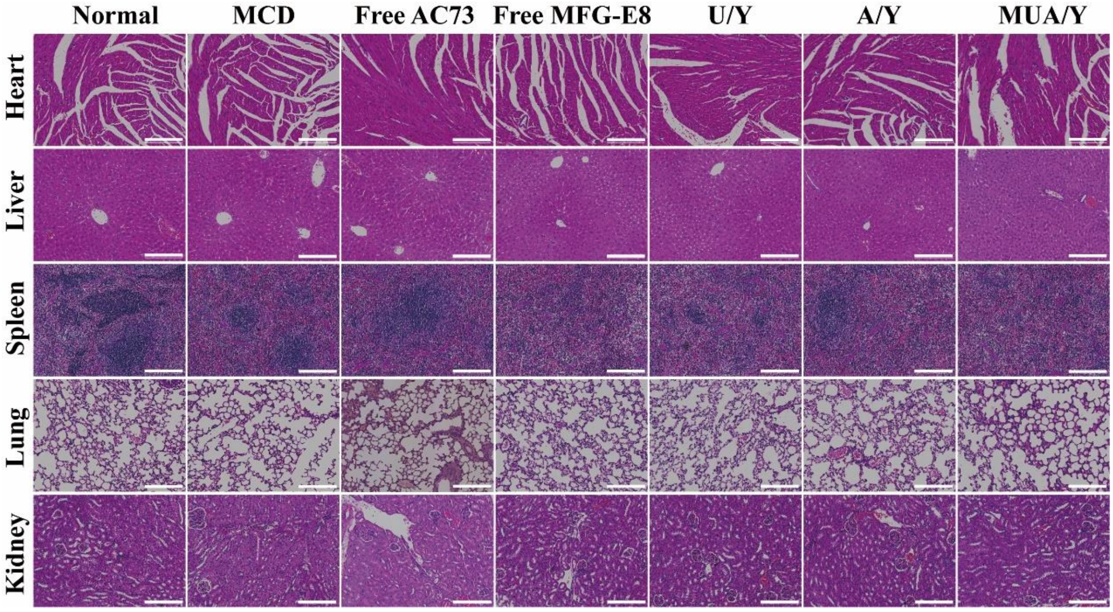

Figure S21. Biosafety evaluation in vivo. H&E assay of the main organs (Heart, Liver, Spleen, Lung, Kidney) after 5 times treatments of different formulations. Scale bar = 200 μm.

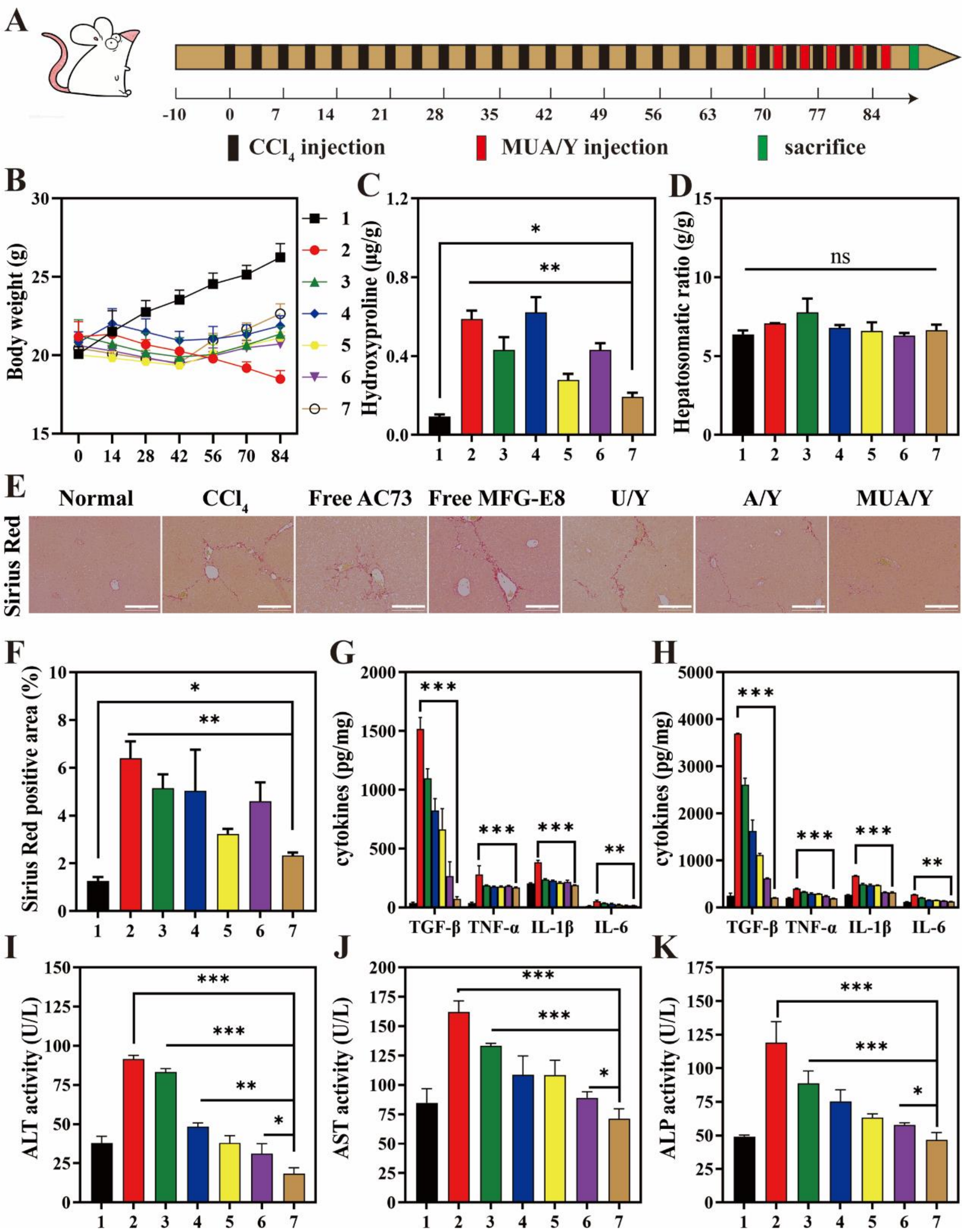

Figure S22. MUA/Y effectively inhibited the progression of CCl_4-induced liver fibrosis. A) Establishment process and treatment strategy of advanced LF model. B) Body weight changes of different groups in the experiment of fibrosis remission, (n = 5). C) The hydroxyproline (Hyp) level in the liver of mice in each treatment group, (n = 3). D) The hepatosomatic ratio of different groups, (n = 3). E) Representative images of Sirius red-stained liver sections, scale bars = 200 μm. F) Semiquantitative analysis of the Sirius-red-positive area, (n = 3). G-H) TGF-β, IL-1β, IL-6, and TNF-α levels in serum and liver tissue, (n = 3). I) Serum ALT activity, (n = 3). J) Serum AST activity, (n = 3). K) Serum ALT activity, (n = 3). 1 means normal mice. 2 means fibrotic mice treated with PBS; 3 means fibrotic mice treated with free AC73; 4 means fibrotic mice treated with free MFG-

E8; 5 means fibrotic mice treated with U/Y; 6 means mice treated with A/Y; 7 means mice treated with MUA/Y; Data were represented as mean ±SD, *****$p < 0.05$, ******$p < 0.01$, *******$p < 0.001$; ns: no significant difference.

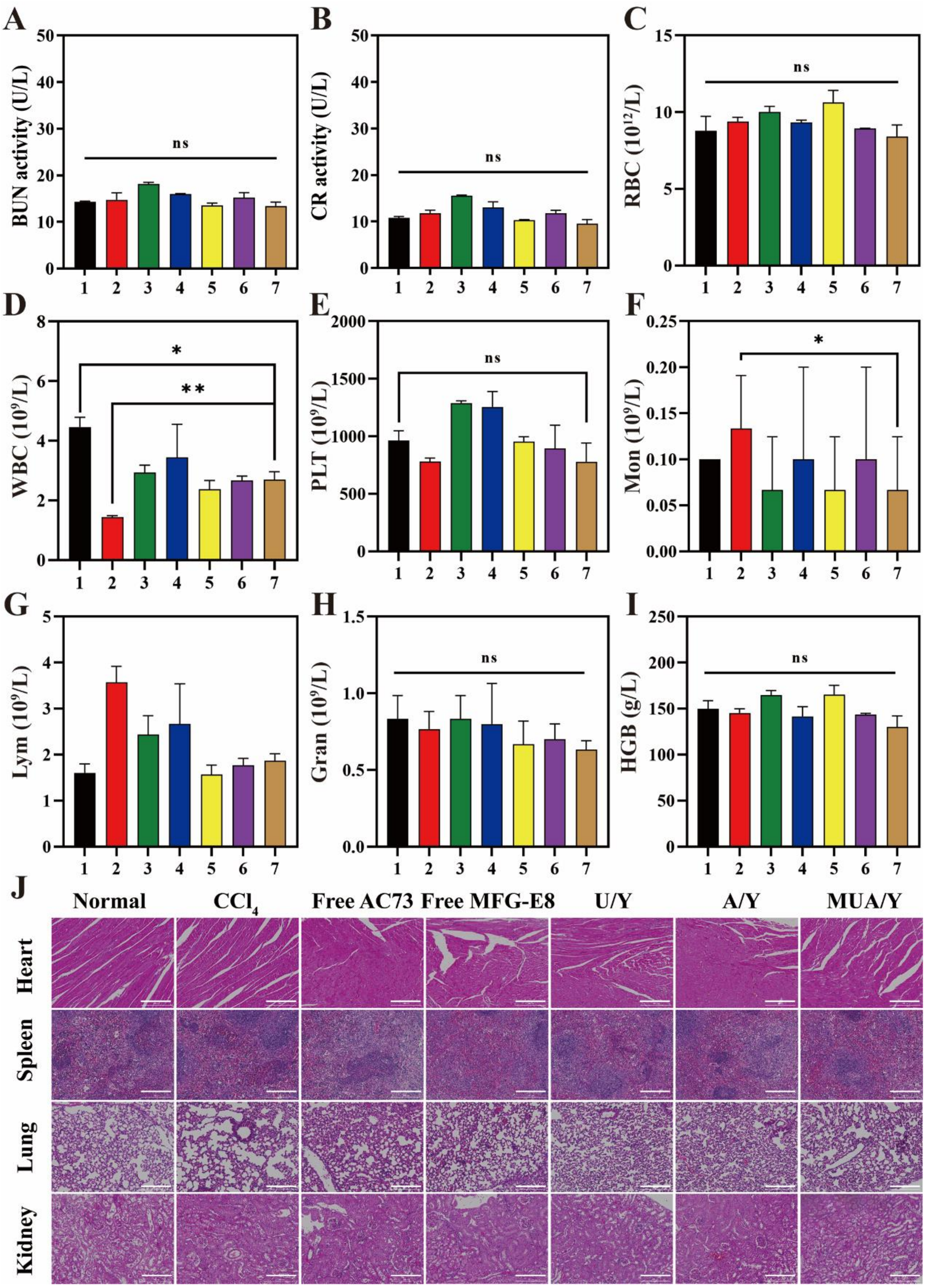

Figure S23. A) Serum BUN activity, (n = 3). B) Serum CR activity, (n = 3). C-I) The levels of physiological markers in different treatment groups, (n = 3). J) Biosafety evaluation *in vivo*. H&E assay of the main organs (Heart, Liver, Spleen, Lung, Kidney) after 5 times treatments of different formulations. Scale bar = 200 μm. 1 means normal mice. 2 means fibrotic mice treated with PBS; 3 means fibrotic mice treated with free AC73; 4 means fibrotic mice treated with free MFG-E8; 5

means fibrotic mice treated with U/Y; 6 means mice treated with A/Y; 7 means mice treated with MUA/Y; Data were represented as mean ±SD, *****$p < 0.05$, ******$p < 0.01$, ns: no significant difference.

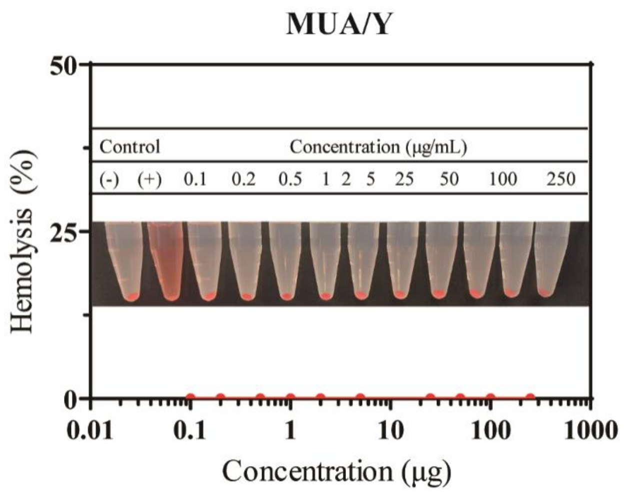

Fig. S24. Hemolysis of MUA/Y lipid nanoparticles at various concentrations on mouse red blood cells. Normal PBS and Triton X-100 solution (10 mg mL^{-1}) were used as negative and positive, respectively.
